# Circumpolar Genetic Structure and Recent Gene Flow of Polar Bears: A Reanalysis

**DOI:** 10.1371/journal.pone.0148967

**Published:** 2016-03-14

**Authors:** René M. Malenfant, Corey S. Davis, Catherine I. Cullingham, David W. Coltman

**Affiliations:** Department of Biological Sciences, University of Alberta, Edmonton, Alberta, Canada; BiK-F Biodiversity and Climate Research Center, GERMANY

## Abstract

Recently, an extensive study of 2,748 polar bears (*Ursus maritimus*) from across their circumpolar range was published in PLOS ONE, which used microsatellites and mitochondrial haplotypes to apparently show altered population structure and a dramatic change in directional gene flow towards the Canadian Archipelago—an area believed to be a future refugium for polar bears as their southernmost habitats decline under climate change. Although this study represents a major international collaborative effort and promised to be a baseline for future genetics work, methodological shortcomings and errors of interpretation undermine some of the study’s main conclusions. Here, we present a reanalysis of this data in which we address some of these issues, including: (1) highly unbalanced sample sizes and large amounts of systematically missing data; (2) incorrect calculation of *F*_*ST*_ and of significance levels; (3) misleading estimates of recent gene flow resulting from non-convergence of the program BayesAss. In contrast to the original findings, in our reanalysis we find six genetic clusters of polar bears worldwide: the Hudson Bay Complex, the Western and Eastern Canadian Arctic Archipelago, the Western and Eastern Polar Basin, and—importantly—we reconfirm the presence of a unique and possibly endangered cluster of bears in Norwegian Bay near Canada’s expected last sea-ice refugium. Although polar bears’ abundance, distribution, and population structure will certainly be negatively affected by ongoing—and increasingly rapid—loss of Arctic sea ice, these genetic data provide no evidence of strong directional gene flow in response to recent climate change.

## Introduction

Polar bears (*Ursus maritimus*) are Holarctic marine mammals that are dependent on sea ice as a platform for mating, reproduction, and locomotion. The southern boundary of their distribution is limited by the extent of the sea ice, which forms the habitat for their primary prey, pagophilic seals such as ringed seals (*Pusa hispida*) and bearded seals (*Erignathus barbatus*). Though long-distance swimming [1] and overland migration [2] are possible, open water, land, and multiyear ice—which is too thick for seals to create breathing holes—generally form barriers to movement and gene flow [3, 4]. Although polar bears have large home ranges [5] and are capable of travelling vast distances [6], gene flow among subpopulations appears to be limited [7]. Currently, 19 management units (MUs) of polar bears are recognized globally, including the Arctic Basin, which is believed to be poor-quality habitat that hinders movement of bears across the area of the North Pole [8]. MUs have been delineated based on radio-telemetry data (primarily of females), hunter tag returns (primarily of males), and genetic data [3, 4, 8–11].

The genetic structure of polar bears has been well characterized in a number of previous studies. The most important of these studies used 16 microsatellites and assignment tests to detect four moderately differentiated genetic clusters across the Arctic, corresponding to the Hudson Bay Complex, the Canadian Arctic Archipelago, the Polar Basin, and Norwegian Bay [4]. Each of these clusters is represented in Canada, and all were recently re-detected in a population genetics study of Canadian polar bears using newly collected samples and thousands of single-nucleotide polymorphisms (SNPs) [12]. Of particular interest is the small, isolated Norwegian Bay MU of the Canadian High Arctic, which is separated from surrounding MUs by thick ice, land, and polynyas [3, 13], and which has been reported as genetically divergent [4, 12] and perhaps phenotypically distinct [3]. Other key population genetics findings include differentiation of Akimiski Island from the rest of Hudson Bay [14–16], east–west differentiation in the Polar Basin [17] (however, cf. [18]), and differentiation in the Canadian Archipelago in the area of the Gulf of Boothia and M’Clintock Channel MUs [19].

In a recent study published in PLOS ONE, Peacock *et al*., 2015 [20] present an analysis based on an impressive dataset of up to 21 nuclear microsatellites and the mitochondrial control region (plus tRNA^Pro^, tRNA^Thr^, and partial cytb) obtained from 2748 and 411 polar bears respectively. Individuals were included from 18 of 19 global MUs (omitting the largely uninhabitable Arctic Basin). Key findings from this study include: (1) a revision of global population genetic structure for polar bears, with three–four major genetic clusters differing somewhat from those that have previously been reported, i.e. [4, 12]: the Canadian Arctic Archipelago, Southern Canada, and the Polar Basin (further subdivided into eastern and western sub-clusters); (2) highly directional recent gene flow into the Canadian Arctic Archipelago from Southern Canada and the Eastern Polar Basin, perhaps due to altered sea-ice conditions caused by climate change, (3) male-biased gene flow, (4) a possible role for the Canadian Arctic Archipelago (and other scattered areas such as the Barents Sea) as interglacial refugia. Most striking among their results, however, is the disappearance of the Norwegian Bay genetic cluster—an important change that is never discussed in Peacock *et al*., 2015 [20].

Upon examination of the article’s methods and supplementary material, we discovered a number of serious errors that call into question the population grouping used in the paper and other conclusions. These include the following (all references to tables and figures are those from Peacock *et al*., 2015 [20]):

- Large amounts of systematically missing data (i.e., genotypes for 5/21 microsatellite loci are missing in at least 6/18 MUs) and differences in sample sizes among MUs that are of two orders of magnitude (S1 Table in [20]).
- Miscalculation of *F*_*ST*_ and other measures of genetic differentiation because of the retention of loci with missing data, such that average pairwise *F*_*ST*_ between all MUs globally is actually negative for microsatellites (–0.03), with values ranging as low as –0.26 (S5 Table; S4 Fig in [20]).
- Bonferroni correction of significance thresholds that incorrectly account for the number of loci rather than the number of tests (S4, S5, S6, S7 Tables in [20]).
- Probable non-convergence of the program BayesAss (S8 Table in [20]; see below for explanation).
- Retention of the M’Clintock Channel, Norwegian Bay, and Viscount Melville MUs in population-level analyses of mitochondrial DNA (mtDNA) despite small sample sizes (i.e., *N* ≤ 3) that are inadequate for estimating haplotype frequencies, and treatment of the Laptev Sea MU as a single subpopulation despite huge geographical discontinuity in sampling and significant deviation from Hardy–Weinberg equilibrium (S1 Table in [20]).

### Non-convergence of BayesAss

The most important conclusion in Peacock *et al*., 2015 [20] is that there has been a recent influx of polar bears into the Canadian Arctic Archipelago from Southern Canada (and the Eastern Polar Basin) in response to recent climate change. They also report a surprising 29-fold difference in directional gene flow from the Eastern Polar Basin to the Western Polar Basin. However, the results given in the Supplementary Material (S8 Table of Peacock *et al*., 2015 [20]) strongly suggest that their BayesAss analysis of recent gene flow failed to converge. Non-convergence is a common problem for BayesAss [21, 22], and non-converged runs often show a bimodal distribution of proportions of non-migrants (*Prop*_*non-mig*_) with some populations having *Prop*_*non-mig*_ < 0.73 and the remainder having *Prop*_*non-mig*_ > 0.9 [21]. Non-convergence is particularly likely when *F*_*ST*_ values are low, and immigration rates may be particularly untrustworthy if they have narrow confidence intervals near one of the prior bounds (i.e., 0 or 1/3) [22]. This is because BayesAss bounds *Prop*_*non-mig*_ between 0.667 and 1 [22, 23]. In S8 Table of Peacock *et al*., 2015 [20], *Prop*_*non-mig*_ (and 95% CIs) are reported for the four genetic clusters as: Eastern Polar Basin = 0.941 (0.888–0.993), Western Polar Basin = 0.678 (0.657–0.699), Canadian Archipelago = 0.699 (0.621–0.777), and Southern Canada = 0.952 (0.912–0.991). These results follow the bimodal distribution described above, and all 95% CIs either overlap the lower bound or are <0.01 from the upper bound. Although it is stated in Peacock *et al*., 2015 [20] that 3–4 runs resulted in similar estimates, this does not indicate that runs converged or that results are accurate, because multiple runs often get trapped near the program’s bounds [21].

To address some of these issues, we reanalysed the original dataset. Because the analyses in Peacock *et al*., 2015 [20] were numerous and wide-ranging, we focused primarily on estimates of contemporary population structure, noting that the generation of contemporary genetic clusters was an important first step for some additional downstream analyses in the original paper, since they formed the groupings between which to test migration, etc. Therefore, this reanalysis may also have implications for some other findings in Peacock *et al*., 2015 [20]. In our opinion, it represents our best estimate to date of the contemporary worldwide population structure of polar bears.

## Materials and Methods

### Nuclear microsatellite data

**Fig 1:**
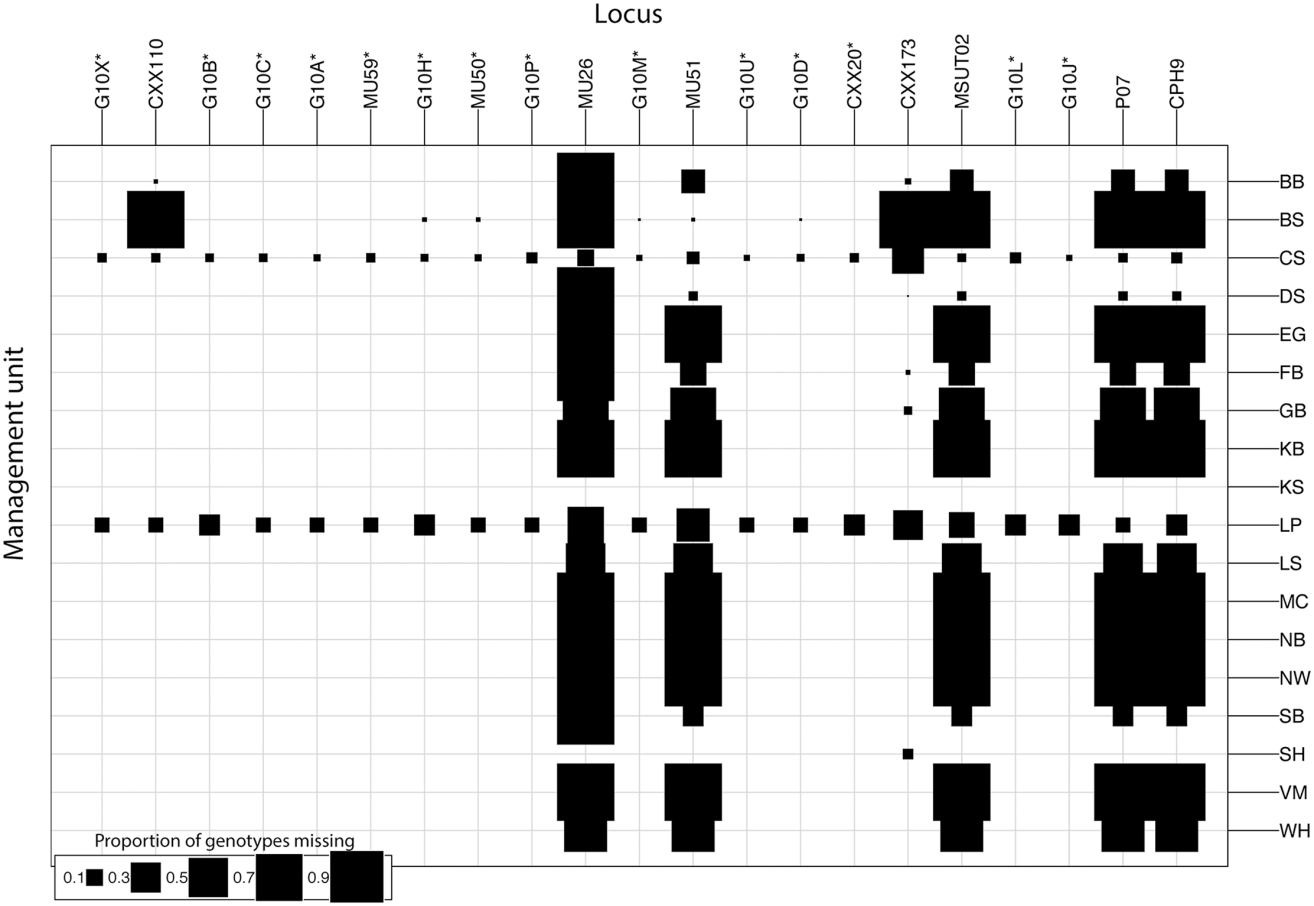
Missing data in Peacock *et al*., 2015 [20]. The size of the square at each management unit–locus intersection is proportional to the amount of missing data at that locus in that management unit. Management unit abbreviations are as in Table 1. Asterisks denote loci that were retained for the reanalysis presented in this paper.

We downloaded the microsatellite genotypes used in Peacock *et al*., 2015 [20] from datadryad.org (doi:10.5061/dryad.v2j1r). Individual-specific information, such as lat–long coordinates, year of sampling, population of sampling, sex, age, etc. are available in Table S11 of Peacock *et al*., 2015 [20]. Methods of DNA extraction, microsatellite genotyping, and genotype quality control are provided in S1 File of Peacock *et al*., 2015 [20]. Microsatellite genotypes were heavily biased towards the Davis Strait (N = 1050) and the Barents Sea (N = 454), Chukchi Sea (N = 266), and Southern Beaufort Sea (N = 233) MUs. Genetic data were compiled from disparate sources (each having been genotyped at different sets of loci), and therefore there are systematic patterns of missing data (Fig 1). Notably, missing data exceeds 80% for marker MU26 and 30% in the Barents Sea MU. Because various programs treat missing data differently (e.g., Structure ignores missing genotypes, GenoDive assigns missing genotypes random values based on allele frequencies, and BayesAss imputes missing genotypes), we reduced the dataset to include only the 14 loci reliably genotyped in all 18 MUs (Fig 1). After an initial analysis, we noticed that Gulf of Boothia was unexpectedly quite divergent from other MUs when using the stepwise-mutation model. We then discovered that the Peacock *et al*., 2015 [20] genotypes for the locus CXX20 were duplicated from the locus CXX110 for many Gulf of Boothia individuals. We replaced these errant CXX20 genotypes with the original genotypes from Paetkau *et al*., 1999 [4].

In Peacock *et al*., 2015 [20], first-degree relatives were excluded based on field data; however, their microsatellite dataset for Davis Strait includes 1050 individuals sampled mostly between 2005 and 2007 (out of an estimated population size of 2158 individuals [24]), and therefore likely still includes many unknown first- and second-degree relatives, the presence of which can cause inaccurate Structure results [25, 26]. Structure also struggles with unbalanced sample sizes [27] and typical pairwise *F*_*ST*_ calculations can be biased by unequal sample sizes as well [28]. Therefore, for MUs having more than 30 microsatellite-genotyped individuals, we used the *sample()* function in R 3.2.0 [29] to randomly select 30 fully genotyped individuals for inclusion in the reduced dataset used in this paper. We used 30 individuals as a cutoff because this was the number used in the last global analysis of population structure [4], and because this number has been shown to be adequate for estimating allele frequencies and *F*_*ST*_ from microsatellite data [30] (however, cf. [31]). One individual from the Laptev Sea had missing data at all loci and was discarded. Our final dataset contained 495 individuals (Table A of S1 File). After filtering all loci and individuals, we checked for Hardy–Weinberg equilibrium in each subpopulation in GenoDive 2.0b27 [32] using Nei’s *G*_*IS*_ statistic [33] (1000 permutations) and for linkage disequilibrium (LD) between loci using Fisher’s method across MUs in GenePop 4.3 [34] (default settings). Unless otherwise indicated, a significance level of *α* = 0.05 was used for all tests, with a Holm correction [35] in the *p*.*adjust()* function of R to account for multiple tests where appropriate.

### Mitochondrial sequence data

We obtained haplotypes from GenBank according to accession numbers and haplotype counts specified in S2 Table of Peacock *et al*., 2015 [20]. The haplotypes UMACR17 and UMACR87 were identical, so we combined these haplotype counts in our dataset. We aligned sequences using MAFFT 7.221 (1PAM/κ = 2 scoring matrix and default settings for auto-strategy; [36]) and after trimming extraneous bases from the ends of the alignment, we found that UMACR88 and UMACR3 were also identical, so we merged these counts as well. We estimated the optimal substitution model under the corrected Akaike information criterion (AICc; [37]) using jModelTest 2.1.7 (default settings; [38, 39]) and calculated summary statistics for mtDNA using Arlequin 3.5.2.2 [40].

### Genetic differentiation, principal components analysis, and AMOVAs

To determine if microsatellites were likely to underestimate population differentiation because of high mutation rates or marker diversity, we tested for a correlation between *G*_*ST*_ and *H*_*S*_ using CoDiDi 1.0 (100,000 permutations; [41]). We calculated pairwise *F*_*ST*_ values [42, 43] between MUs, as well as AMOVAs [43] using GenoDive. We also calculated pairwise *R*_*ST*_ [43, 44] using SPAGeDi 1.4b [45]; however, these results are not presented because an allele-size permutation test (10,000 permutations; [46]) suggested that microsatellite allele sizes were uninformative. To explore the data, we performed a principal components analysis (PCA) of individual genetic variation using adegenet 1.4–2 (centred and scaled, missing data set to mean; [47, 48]). To examine the robustness of our primary conclusion (i.e., the divergence of Norwegian Bay) to the 30-individual sampling process we used to generate our reduced dataset, we also plotted PCAs for 100 additional randomly generated subsamples of the full dataset. We generated a population tree using the recommendations for the infinite-allele model in Takezaki and Nei, 1996 [49]: we estimated the topology of the tree with unweighted pair-group method with arithmetic mean (UPGMA) in POPTREEW [50] using Nei’s *D*_*A*_ [51], then we unrooted the tree and estimated branch lengths using Nei’s standard distance (*D*_*S*_) [52] using non-negative least squares in phangorn 1.99–13 [53].

For mtDNA, we calculated pairwise *F*_*ST*_ and *Φ*_*ST*_ values (and their corresponding AMOVAs [42]) using Arlequin. For *Φ*_*ST*_ calculations, distances between haplotypes were calculated using the Tamura & Nei substitution model [54] with gamma-distributed rate heterogeneity (*α* = 0.021), which was determined as the optimal model of evolution under the AICc. Significance of all pairwise measures was assessed using 10,000 permutations. We also conducted exact tests of population differentiation in GenePop for microsatellites and in Arlequin for mtDNA (default settings). Significance of AMOVAs was not tested because of circularity in the logic of doing so for pre-clustered groups [55]. Pairwise *F*_*ST*_ values for microsatellites and pairwise *Φ*_*ST*_ values for mtDNA were compared with the expectation of $F_{ST}(nu)=1-e^{0.25\times \text{ln}[1-F_{ST}(mt)]}$ [56], as was used in Peacock *et al*., 2015 [20], to determine whether polar bears exhibit male-biased gene flow.

### Clustering methods and isolation by distance

The settings used for Structure analysis (e.g., number of repetitions, length of burn-in, priors) were not given in Peacock *et al*., 2015 [20]. We followed the recommendations of Gilbert *et al*., 2012 [57]: 20 independent runs of 200,000 iterations (incl. 100,000 burn-in iterations) using the correlated allele frequencies model with no location prior using Structure 2.3.4 [58, 59]. Runs were clustered and averaged using CLUMPAK 1.1 (default settings; [60]), and support for *K*-values was generated in CLUMPAK’s “Best *K*” feature using the Evanno method [61] and the Pritchard method [62]. As has been recommended in the case of low genetic differentiation [63], we compared the output from Structure with output from BAPS 6.0 using its non-spatial admixture mode (*K*_*max*_ = 20; *N*_*min*_ = 5; *N*_*it*_ = 100; *N*_*ref_ind*_ = 200; *N*_*ref_it*_ = 10; [64, 65]). To infer genetic clusters for individuals used in the original study but not included in our reduced dataset, we used trained clustering in BAPS [65–67], using non-admixed individuals from each genetic cluster as the training set. We also grouped MUs using AMOVA-based *K*-means clustering in GenoDive for *K* = 6, which was found to be the optimal *K*-value in Structure analyses. Finally, to confirm the hierarchical structure (i.e., east–west differentiation) that we detected within the Canadian Arctic Archipelago and the Polar Basin, we ran Structure on the full set of samples collected within each of these MUs using LOCPRIOR = 1 to improve the power to detect weak differentiation [68].

Isolation by distance (IBD) can confound clustering analyses [69]. Because the optimal Structure results for *K* = 6 showed an east–west cline in *Q*-values across the Polar Basin, and because there was a large sampling discontinuity in the middle of this cline (i.e., in the Laptev Sea MU), we suspected that one of these two clusters may have been spuriously generated by IBD. To test for IBD across this region, we performed a Mantel test between genetic distances [70] and geographical distances (calculated in SPAGeDi) for all individuals that were highly assigned (i.e., CLUMPAK-averaged *Q* ≥ 0.7) to either the Eastern or Western Basin clusters (N = 62). To determine if IBD alone might be responsible for observed east–west genetic clustering in the Basin, we performed a partial Mantel test of association between a matrix of genetic distances and a model matrix denoting whether each pair of individuals belonged to the same genetic cluster (= 0) or not (= 1), while conditioning on geographical distances (cf. [71]). Both tests were conducted in vegan 2.2–1 [72], using 10,000 permutations to test for significance.

### Migration rates

Using BayesAss 3.0.3 [73], we attempted to re-estimate rates of gene flow between five of our six regions (Eastern & Western Polar Basin, Eastern & Western Archipelago, Hudson Complex) and—for comparison—between three of the four major regions identified as optimal by Paetkau *et al*., 1999 [4] and in our *K* = 4 Structure results (i.e., Polar Basin, Archipelago, Hudson Complex). We omitted Norwegian Bay from both of these runs because its small sample size might result in non-converged estimates [21], and before running BayesAss, we used assignment tests in GenoDive to remove any significant (default settings, 1000 permutations) immigrants from Norwegian Bay found in other MUs. Because our dataset of ~30 samples per MU does not accurately reflect differences in MU population size that would affect gene-flow estimates when MUs were merged into regions, we generated balanced subsets using the sampling regimes shown in Table D of S1 File. Individuals were selected for inclusion in these subsets blindly (i.e., without viewing their genetic cluster membership) while attempting to obtain geographical balance and high sample sizes of 100–150 individuals per region, which have been shown to be correlated with the probability of convergence [21]. For direct comparison with Peacock *et al*., 2015 [20], we also generated a balanced subset corresponding to their four regions (Eastern & Western Polar Basin, Archipelago, Hudson Complex). For all BayesAss runs, we followed the recommendations of Faubet *et al*., 2007 [22] (i.e., ten runs with different random seeds, *N*_*it*_ = 21,000,000, *N*_*burn-in*_ = 2,000,000, sampling interval = 2,000), and we used the Bayesian deviance (as calculated in the *calculateDeviance*.*R* script from Meirmans, 2014 [21]) to select the best run. Convergence of parameter estimates in these best runs was also assessed by manual examination of trace files, and by using the Heidelberger-and-Welch diagnostics [74] in boa 1.1.8–1 [75]. Significant differences in proportions of immigrant ancestry were assessed using non-overlapping 95% CIs. To ensure that we were not unintentionally broadening CIs by using only 14 loci, we also performed runs for all datasets including all 21 loci. Finally, to test whether the placement of the Laptev Sea MU (which straddles the apparent boundary between the Western and Eastern Polar Basin clusters) affected our results, we considered a run that excluded this MU entirely.

## Results

### Nuclear microsatellite and mitochondrial DNA statistics

We found that one MU, the Laptev Sea, exhibits significant heterozygote deficiency (*G*_*IS*_ = 0.15, *P* < 0.001; Table 1), likely because of a Wahlund effect [76] caused by discontinuous sampling in this MU: there is a >1,400 km gap between western and eastern Laptev Sea samples. Because subpopulation deviation from Hardy–Weinberg equilibrium affects *F*-statistics, we followed Paetkau *et al*., 1999 [4] in excluding the Laptev Sea from all MU-based analyses such as LD and pairwise *F*_*ST*_. For AMOVAs and BayesAss analyses of gene flow among major genetic clusters, we apportioned the Laptev Sea MU’s eastern and western samples into the eastern and western Polar Basin clusters, respectively.

**Table 1:** Genetic diversity statistics for 18 management units of polar bears. For microsatellite data, a maximum of 30 individuals have been retained from each management unit from the original dataset of 2,748 individuals, and only the 14 loci indicated in Fig 1 have been used. Molecular diversity indices for mitochondrial DNA were calculated in Arlequin using pairwise differences with no gamma correction.

	Nuclear microsatellites							Mitochondrial DNA				
Management unit (abbr.)	N	YoC	*K*	*%*_*Miss*_	*H*_*O*_	*H*_*E*_	*G*_*IS*_	N	YoC	*K*	*h*	*π*
Baffin Bay (BB)	30	2003	6.43	0	0.74	0.73	–0.01	30	2007	11	0.88	0.0059
Barents Sea (BS)	30	2000	6.36	0	0.66	0.66	0.00	30	1998	13	0.90	0.0057
Chukchi Sea (CS)	30	1997	7.00	0	0.68	0.70	0.02	35	2000	17	0.93	0.0061
Davis Strait (DS)	30	2006	6.71	0	0.67	0.69	0.02	121	2006	21	0.87	0.0039
East Greenland (EG)	30	1990	6.50	0	0.66	0.67	0.02	–		–	–	–
Foxe Basin (FB)	30	2002	6.29	0	0.71	0.69	–0.03	26	2008	6	0.71	0.0028
Gulf of Boothia (GB)	30	2001	6.29	0	0.70	0.72	0.02	16	2008	6	0.68	0.0034
Kane Basin (KB)	30	1994	6.43	0	0.73	0.72	–0.01	–		–	–	–
Kara Sea (KS)	17	1994	5.36	0	0.62	0.64	0.04	17	1994	7	0.84	0.0044
Laptev Sea (LP)	14	2000	5.79	2.6	0.59	0.70	**0.15**	14	2000	11	0.97	0.0061
Lancaster Sound (LS)	30	2002	6.57	0	0.74	0.71	–0.03	34	2007	11	0.86	0.0066
M’Clintock Channel (MC)	14	1996	5.50	0	0.71	0.69	–0.03	2	2008	1	0	0
Northern Beaufort Sea (NB)	30	1989	6.79	0	0.68	0.69	0.00	–		–	–	–
Norwegian Bay (NW)	30	1995	6.21	0	0.68	0.69	0.01	3	2008	1	0	0
Southern Beaufort Sea (SB)	30	2001	6.79	0	0.65	0.68	0.04	30	1997	15	0.94	0.0073
Southern Hudson Bay (SH)	30	2008	5.86	0	0.66	0.66	0.00	23	2008	8	0.58	0.0019
Viscount Melville Sound (VM)	30	1991	6.29	0	0.64	0.66	0.03	3	2008	1	0	0
Western Hudson Bay (WH)	30	1998	6.14	0	0.65	0.67	0.02	27	2007	9	0.86	0.0047

N, number of individuals genotyped; YoC, mean year of sample collection; *K*, number of alleles; *%*_*Miss*_, percentage of genotypes missing; *H*_*O*_, observed heterozygosity; *H*_*E*_, expected heterozygosity; *G*_*IS*_, heterozygosity-based estimator of individual-level inbreeding within a subpopulation; *h*, haplotype diversity; *π*, nucleotide diversity. In the *G*_*IS*_ column, boldface denotes significant deviation from Hardy–Weinberg equilibrium.

No locus deviated significantly from Hardy–Weinberg equilibrium. Two pairs of loci were in significant LD (G10B–G10J, G10B–G10X); however, both had *P* = 0 in one MU, which causes problems for Fisher’s method [77], and neither pair is located on the same genomic scaffold [12]. Even if the scaffolds were contiguous within a chromosome, these markers would be separated by >5 Mb, and at these distances, LD is negligible in polar bears [12]. Therefore, we assumed these were false positives, and we elected to keep all 14 microsatellite markers for subsequent analysis. *H*_*S*_ and *G*_*ST*_ were not significantly negatively correlated (*r* = –0.008, *P* = 0.467), indicating that microsatellites were unlikely to underestimate population differentiation because of high mutation rates or marker diversity. Three MUs had inadequate sampling (i.e., N ≤ 3, *k* = 1) to accurately determine mitochondrial haplotype frequencies: namely, M’Clintock Channel, Norwegian Bay, and Viscount Melville Sound. Therefore, we excluded these MUs in pairwise population comparisons and AMOVAs of mtDNA.

### Clustering of individuals and management units

**Fig 2:**
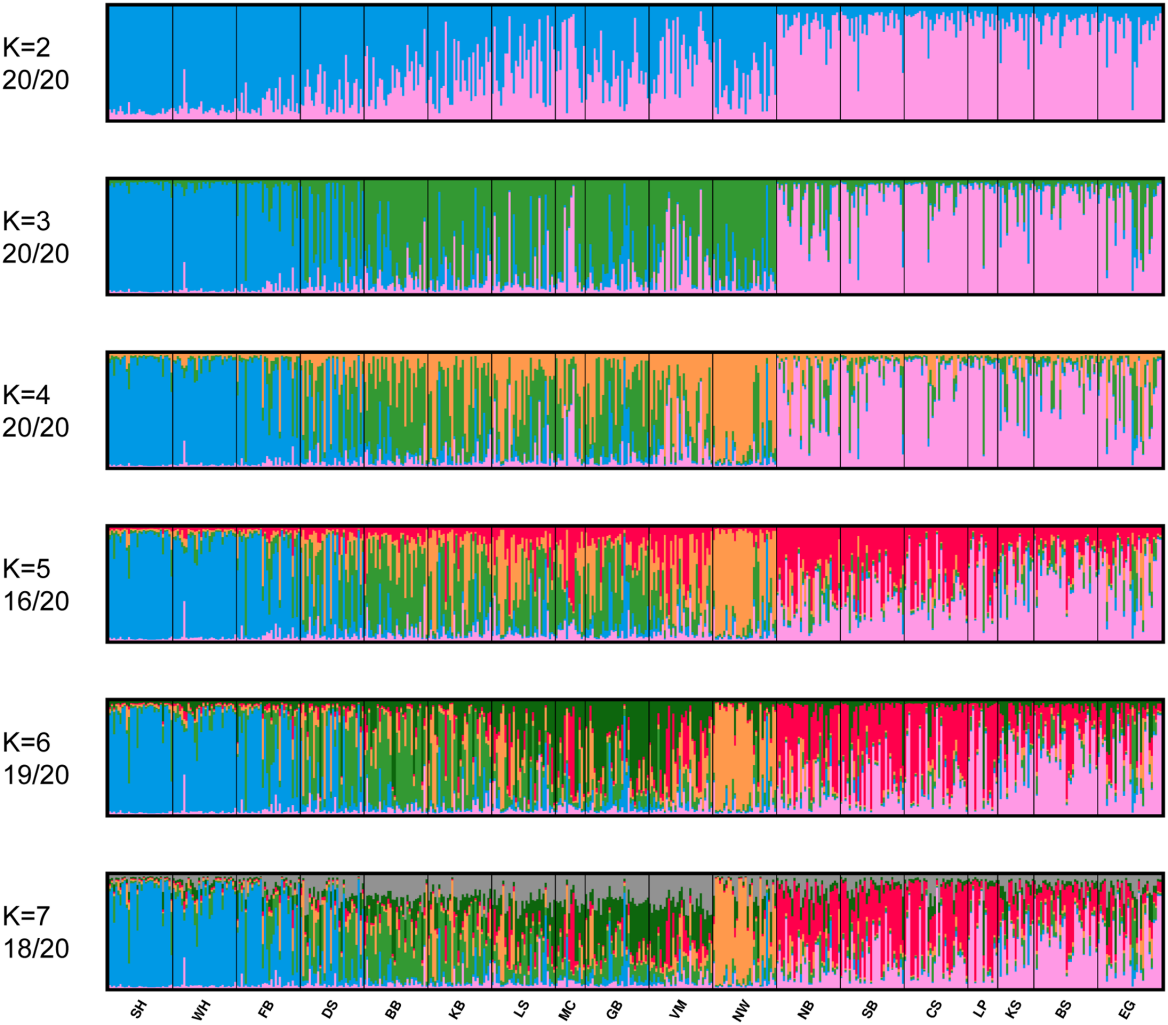
CLUMPAK-averaged Structure outputs for 20 independent runs of *K* = 2–7, which were clustered and averaged using CLUMPAK. Numbers under each K-value indicate the proportion of runs that converged to the solution presented. Minority modes supported by at least two runs are provided in Fig A in S1 File. Management unit abbreviations are as in Table 1.

**Fig 3:**
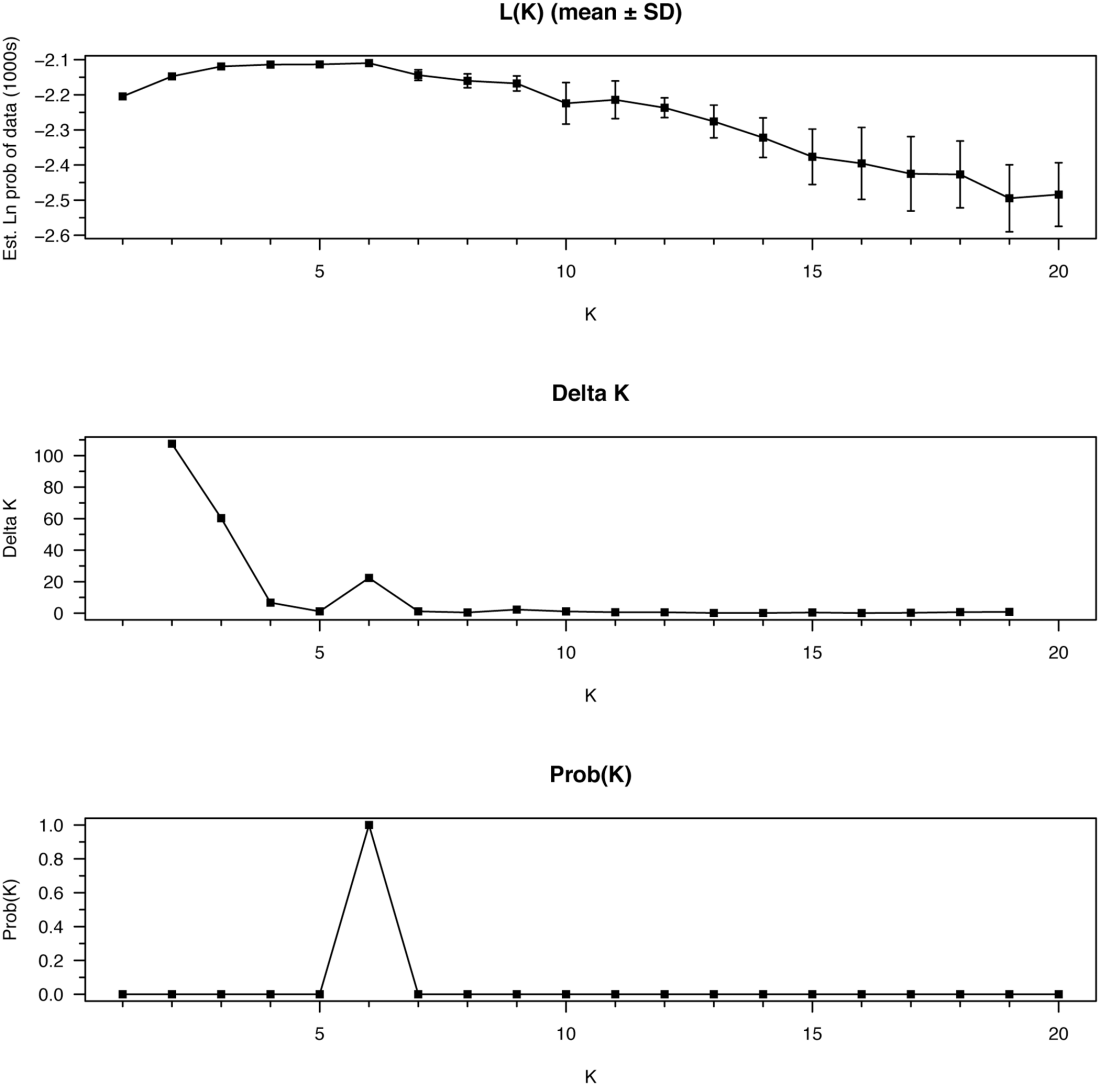
(a) Structure output of mean likelihood ± SD calculated from 20 independent runs for each value of *K* from 1 to 20. (b) *ΔK* calculated using the Evanno method in CLUMPAK. (c) Probability of K calculated using the Pritchard method in CLUMPAK.

CLUMPAK-averaged admixture plots for *K* = 2–7 are shown in Fig 2. Progressively, they show the addition of clusters that largely correspond to the following, with some apparent admixture and migration: *K* = 2: the Polar Basin, *K* = 3: the Canadian Arctic Archipelago, *K* = 4: Norwegian Bay, *K* = 5: west–east differentiation in the Polar Basin, *K* = 6: west–east differentiation in the Canadian Arctic Archipelago, *K* = 7: apparent noise. Although the Evanno *ΔK* method selected *K* = 2 (Fig 3b), likelihood was maximized at *K* = 6 (Fig 3a)—this was the number of clusters preferred using the Pritchard method (Fig 3c), and there was also a small peak in *ΔK* at this value.

The six genetic clusters we detected correspond roughly to: the Hudson Complex (incl. Labrador), the Eastern Archipelago, the Western Archipelago, Norwegian Bay, the Eastern Polar Basin, and the Western Polar Basin. Because these results were geographically defensible and corresponded roughly with previously discovered genetic structure in the Archipelago [4] and across the Polar Basin [17], we accepted *K* = 6 for our Structure analysis. Regional Structure analyses using the full dataset and LOCPRIOR = 1 also detected east–west differentiation within the Archipelago and within the Basin, with possible additional clusters present in the Gulf of Boothia and in the Chukchi Sea (Fig B in S1 File). GenoDive clustering of MUs for *K* = 6 reached similar conclusions as Structure (cf. shaded areas in Table 2), splitting the Archipelago into Eastern (KB, BB, northern DS) and Western (VM, GB, MC, LS) clusters and splitting the Polar Basin into Eastern (EG, KS, BS, eastern LP) and Western (SB, NB, CS, western LP) clusters.

**Table 2:** Pairwise *F*_*ST*_ values for nuclear microsatellites (below diagonal) and pairwise *Φ*_*ST*_ values for mitochondrial DNA (above diagonal); significant values are bolded. MU abbreviations are as in Table 1. Solid lines demarcate the four major clusters discovered by Paetkau *et al*., 1999 [4], which correspond to our Structure results for *K* = 4. From left to right, these are: the Hudson Complex, the Canadian Arctic Archipelago, Norwegian Bay, and the Polar Basin. Dotted lines denote the west–east clusters within the Basin and the Archipelago detected by *K*-means clustering in GenoDive. These six clusters include additional east–west substructure within the Archipelago and within the Polar Basin. DS is an admixture zone showing affinity for both Hudson Complex and the Archipelago, with southern samples tending to belong to the Hudson Complex cluster and northern samples tending to belong to the Eastern Archipelago cluster. LP has been excluded from all comparisons because it deviates significantly from Hardy–Weinberg equilibrium. For mitochondrial DNA, MC, VM, and NW were omitted because sample sizes were too small (i.e., N ≤ 3, *k* = 1) to accurately estimate haplotype frequencies.

	**SH**	**WH**	**FB**	**DS**	**BB**	**KB**	**LS**	**GB**	**MC**	**VM**	**NW**	**NB**	**SB**	**CS**	**LP**	**KS**	**BS**	**EG**
**SH**	–	0.11	0.04	0.11	0.06		0.15	0.00					0.16	0.05		**0.44**	0.12	
**WH**	0.01	–	0.05	**0.14**	**0.19**		**0.30**	0.13					**0.31**	**0.23**		**0.47**	**0.25**	
**FB**	0.01	0.01	–	0.06	0.06		0.19	0.05					**0.23**	0.13		**0.47**	0.14	
**DS**	**0.03**	**0.03**	**0.01**	–	**0.15**		**0.33**	0.13					**0.34**	**0.17**		**0.47**	**0.23**	
**BB**	**0.05**	**0.05**	**0.03**	**0.01**	–		0.02	0.02					0.10	0.06		0.20	0.03	
**KB**	**0.05**	**0.05**	**0.04**	**0.02**	0.00	–												
**LS**	**0.05**	**0.05**	**0.04**	**0.02**	0.01	0.01	–	0.09					0.02	0.09		0.13	0.10	
**GB**	**0.05**	**0.04**	**0.03**	**0.02**	0.01	**0.02**	0.00	–					0.10	0.03		**0.37**	0.10	
**MC**	**0.06**	**0.05**	**0.04**	**0.03**	0.02	0.01	0.00	0.01	–									
**VM**	**0.07**	**0.05**	**0.05**	**0.04**	**0.03**	**0.02**	0.00	**0.02**	0.01	–								
**NW**	**0.07**	**0.06**	**0.05**	**0.04**	**0.03**	**0.03**	**0.02**	**0.04**	**0.04**	**0.03**	–							
**NB**	**0.09**	**0.08**	**0.06**	**0.05**	**0.04**	**0.04**	**0.02**	**0.04**	**0.03**	**0.03**	**0.05**	–						
**SB**	**0.10**	**0.08**	**0.07**	**0.06**	**0.04**	**0.05**	**0.03**	**0.04**	**0.03**	**0.04**	**0.07**	0.01	–	0.07		0.15	0.11	
**CS**	**0.10**	**0.09**	**0.07**	**0.07**	**0.04**	**0.05**	**0.04**	**0.05**	**0.03**	**0.04**	**0.06**	0.00	0.00	–		**0.18**	0.08	
**LP**															–			
**KS**	**0.09**	**0.07**	**0.06**	**0.05**	**0.04**	**0.04**	**0.03**	**0.05**	0.02	**0.03**	**0.06**	0.01	0.01	0.01		–	0.07	
**BS**	**0.10**	**0.08**	**0.07**	**0.05**	**0.04**	**0.04**	**0.04**	**0.05**	**0.03**	**0.05**	**0.07**	**0.02**	**0.02**	**0.02**		0.01	–	
**EG**	**0.09**	**0.08**	**0.06**	**0.05**	**0.03**	**0.03**	**0.03**	**0.05**	**0.03**	**0.04**	**0.05**	**0.02**	**0.02**	**0.02**		0.01	0.00	–

**Fig 4:**
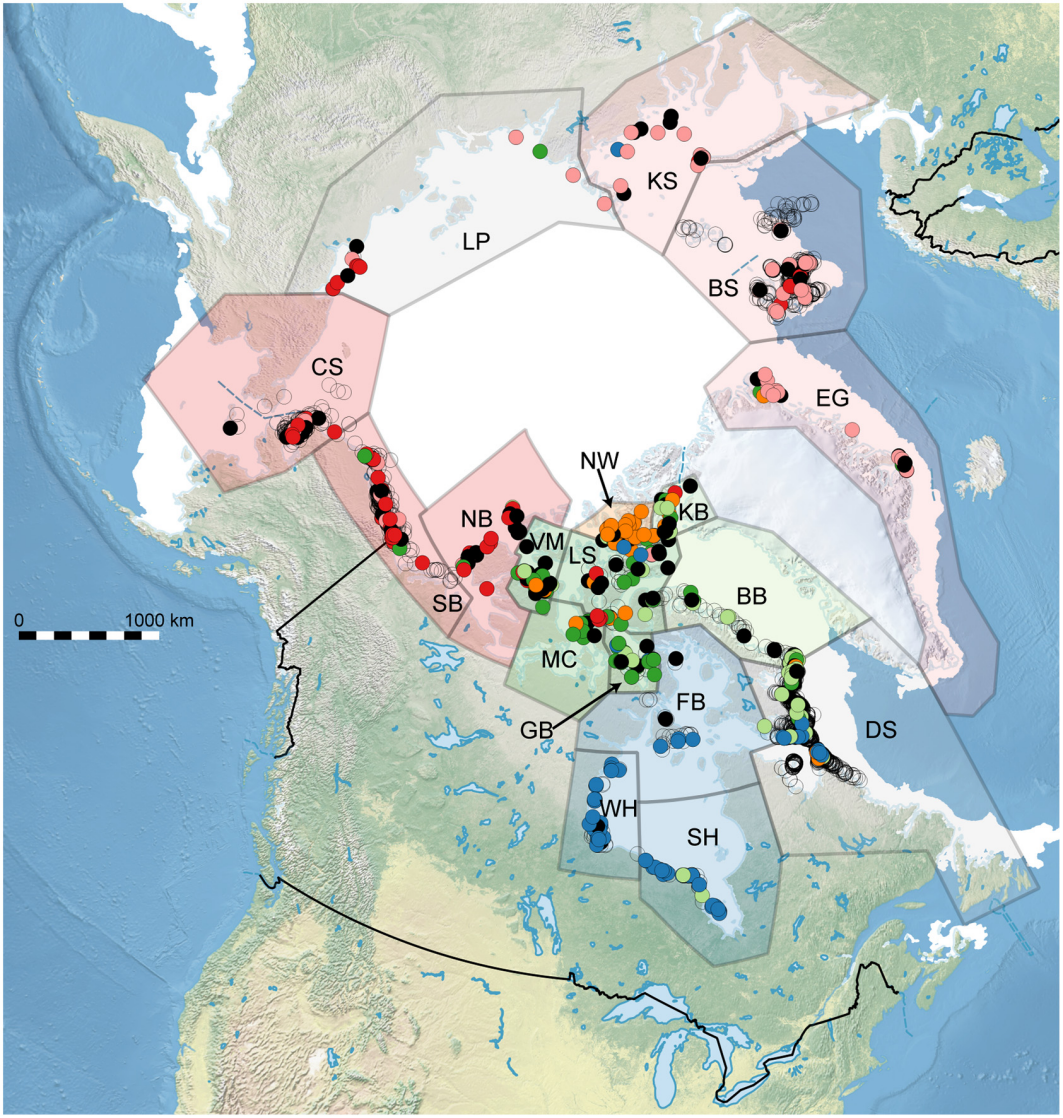
Sampling locations for 476 of the 495 polar bears used in this analysis; the remainder did not have lat–long coordinates. Individuals are colour-coded by genetic cluster similarly to the colour scheme for *K* = 6 in Fig 2. Black samples are unassigned (i.e., *Q*_*max*_ < 0.5). Uncoloured individuals are those that were used in the original study but were not included in our random subset of 30 individuals per management unit; their predicted cluster memberships based on BAPS trained clustering are shown in Fig C of S1 File. Management unit abbreviations are as in Table 1. Approximate sea ice extent during the breeding season is shown using measurements for April 15, 2008 [78], though there is great spatial heterogeneity in sea ice thickness and concentration, as well as great intra-seasonal and inter-annual variability. Note that this map (and the data) does not reflect a 2014 boundary change between NB and SB made by the territorial governments and the co-management boards with management authority for these two subpopulations, because it has not yet been evaluated by the IUCN Polar Bear Specialist Group.

There is significant IBD across the Polar Basin (Mantel test: *r* = 0.2354, *P* < 0.0001), though genetic clustering remained marginally significant after accounting for IBD (partial Mantel test: *r* = 0.06391, *P* = 0.039). Therefore, we decided to retain both the Eastern and the Western Basin clusters, though we note that traversable distances between individuals in this region will be underestimated by SPAGeDi if it calculates distances over the poor-quality Arctic Basin MU. Results are mapped in Fig 4.

Mixture analysis in BAPS found *K* = 6 as being optimal; however, one of these clusters contained only a single individual with missing data at four loci, perhaps indicative of the unexpected effects that missing data can have upon such methods. This single-individual cluster was discarded prior to admixture analysis. The remaining clusters were: the Hudson Complex, the Eastern and Western Canadian Arctic Archipelago, the Polar Basin, and Norwegian Bay. Results of the BAPS admixture analysis based on these five clusters is found in Fig C of S1 File; they differ from the optimal Structure results for *K* = 6 in that there is less admixture and no distinction of the Eastern/Western Polar Basin. Trained clustering in BAPS using *K* = 5 gave sensible estimates of genetic-cluster membership for all individuals not included in our main study (Fig D of S1 File).

### Population differentiation

**Fig 5:**
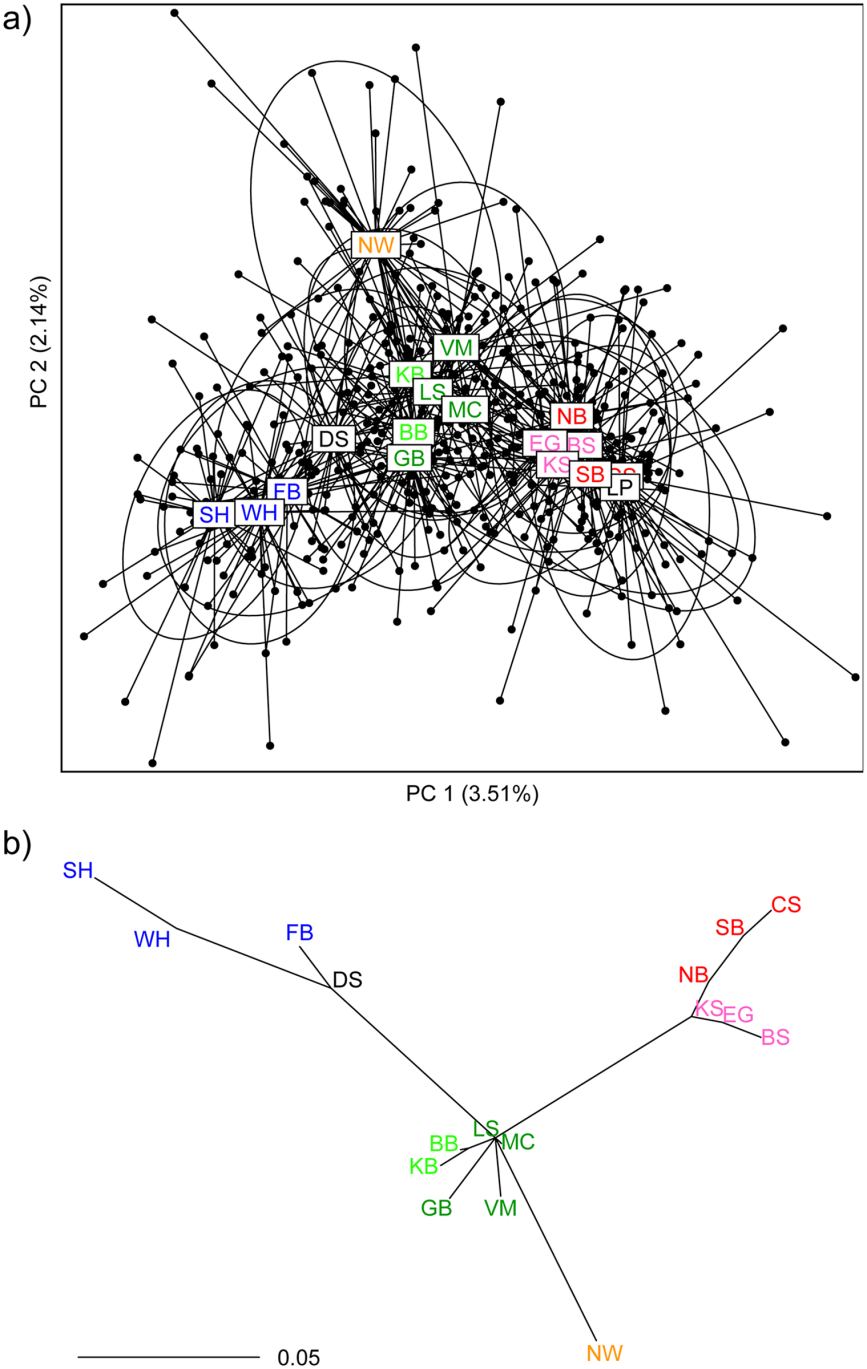
(a) Principal component analysis of genetic variation. Each point represents an individual; each individual is connected to a label indicating the centroid of the management unit in which it was sampled. The inertia ellipse for each management unit contains approximately two-thirds of all individuals sampled there. (b) Population tree. The scale bar indicates Nei’s standard distance; branch lengths were estimated using non-negative least squares, and the tree has an *R*^2^ [79] of 0.903. Samples from the Laptev Sea (LP) have been excluded in (b) because a large spatial discontinuity in sampling in this management unit resulted in it being significantly out of Hardy–Weinberg equilibrium. Management unit abbreviations are as in Table 1 and are coloured as in Fig 4.

Our PCA and population tree reveal four broad groupings of MUs that correspond to the clusters identified by our Structure results for *K* = 4 and by Paetkau *et al*., 1999 [4] (Fig 5): the Polar Basin (CS, SB, BS, NB, EG, KS), the Archipelago (MC, VM, LS, BB, KB, GB), Norwegian Bay (NW), and the Hudson Complex (DS, FB, WH, SH). These four groupings were also seen in most of our other 100 randomly resampled subsets of individuals (Figs E and F of S1 File). The six genetic clusters selected by GenoDive explain ~3.9% of the nuclear genetic variation and ~9.4% of genetic variation in mtDNA. MU designations within clusters explain 0.8% and 6.0% for microsatellites and mtDNA respectively (Tables 3, 4). Overall, MUs were slightly to moderately differentiated (average pairwise microsatellite *F*_*ST*_ = 0.04). Tests of pairwise population differentiation revealed many significant differences between major genetic clusters, but few significant differences within clusters. In total, 121/136 (≈89%) of population pairs were significantly differentiated after a Holm correction based on nuclear *F*_*ST*_, genic, or genotypic differentiation (compared to only 20% in Peacock *et al*., 2015 [20], who also included tests for *R*_*ST*_). Importantly, all tests of genetic differentiation show that Norwegian Bay is significantly differentiated from all other MUs (Table 2, Table B of S1 File). Though Gulf of Boothia differed significantly from most nearby MUs in tests of genotypic and genic differentiation of nuclear markers (Table B of S1 File), it was not as well differentiated from other members of the Western Archipelago using pairwise *F*_*ST*_ or *Φ*_*ST*_ (Table 2) or tests of haplotypic differentiation for mtDNA (Table C of S1 File).

**Table 3:** Hierarchical analysis of molecular variance (AMOVA) for nuclear microsatellites among management units within the six genetic clusters identified in this paper and shown in Table 2. For this analysis, we followed Peacock *et al*., 2015 [20] by including northern Davis Strait in the Eastern Archipelago cluster and southern Davis Strait in the Hudson cluster. Western Laptev was included in the Western Basin cluster and Eastern Laptev was included in the Eastern Basin cluster. However, results did not differ significantly when the Laptev Sea and Davis Strait MUs were excluded entirely.

Source of variation	% variance	*F*-statistic	*F*-value
Within individuals	94.32%	*F*_*IT*_	0.057
Among individuals in MUs	1.07%	*F*_*IS*_	0.011
Among MUs in clusters	0.79%	*F*_*SC*_	0.008
Among clusters	3.87%	*F*_*CT*_	0.039

**Table 4:** Hierarchical analysis of molecular variance (AMOVA) for mitochondrial DNA among management units within the six genetic clusters identified in this paper and shown in Table 2. Note that many management units (incl. the entire Norwegian Bay cluster) were excluded entirely from this AMOVA because of inadequate sampling. Because we lacked sample location information for downloaded haplotypes, we were unable to split Davis Strait or the Laptev Sea into northern/southern or eastern/western samples; therefore, these MUs were removed for this calculation in addition to the MUs that were removed for low sample sizes in Table 2.

Source of variation	% variance	*Φ*-statistic	*Φ*-value
Among individuals in MUs	84.54%	*Φ*_*ST*_	0.155
Among MUs in clusters	6.04%	*Φ*_*SC*_	0.067
Among clusters	9.43%	*Φ*_*CT*_	0.094

### Recent gene flow and sex-biased dispersal

**Fig 6:**
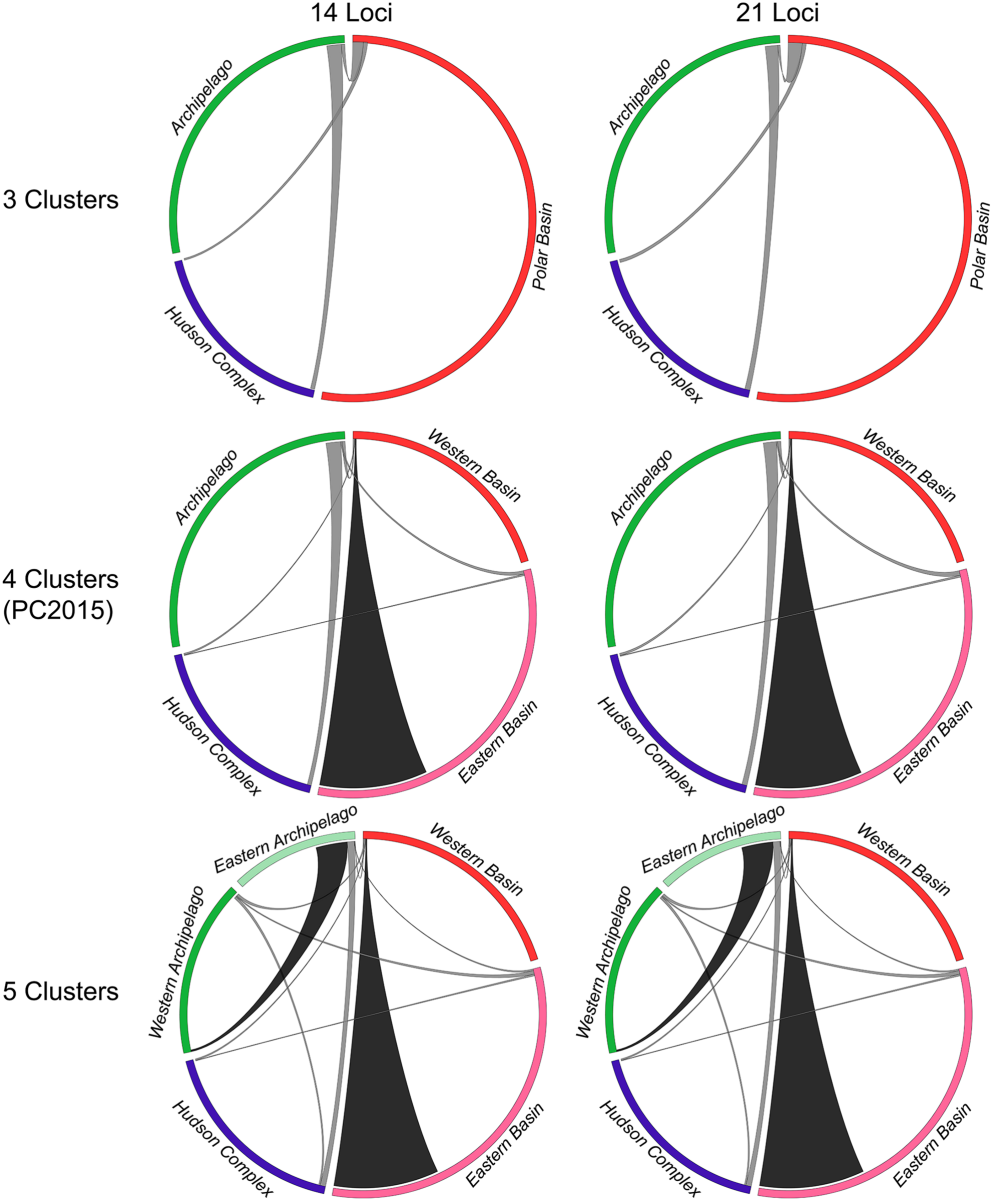
Circos plots of gene flow using 14 or 21 loci among: three clusters corresponding to our Structure results for *K* = 4 (excl. Norwegian Bay samples), four clusters identified in Peacock *et al*., 2015 [20] (excl. Norwegian Bay samples), and five clusters corresponding to our Structure results for *K* = 6 (excl. Norwegian Bay samples). Segment colours are as in Fig 4 and are sized proportionally to the population size estimates in Table D of S1 File, though polar bear population sizes are estimated with very broad confidence intervals, particularly in the Polar Basin, where reliable estimates are not available for most MUs. The width of each ribbon where it meets a segment on the circumference indicates the proportion of migrants into (but not out of) each region. Black ribbons are significantly directional based on non-overlapping 95% CIs of immigration rates; grey ribbons are not significantly directional.

All “best” BayesAss runs for each population grouping (selected based on the deviance) were at stationarity after burn-in, according to Heidelberger-and-Welch diagnostics. Estimates were surprisingly robust to large amounts of systematically missing data, as results for 14 loci and 21 loci were nearly identical in terms of means and confidence intervals (Fig 6); however, because runs for 14 loci had larger effective sample sizes, we discuss these results below. All runs gave highly similar estimates of gene flow among the Polar Basin, the Hudson Complex (incl. Labrador), and the Canadian Arctic Archipelago. In no case was there a significant difference in the proportion of migrants into any of these populations. Within these major clusters, BayesAss suggested highly directional gene flow from the Western Polar Basin into the Eastern Polar Basin, and from the Western Archipelago into the Eastern Archipelago; however, these highly directional estimates are likely untrustworthy, as discussed below. Immigration rates and proportions of non-migrant ancestry are given in Tables E–G of S1 File. Exclusion of the Laptev Sea MU did not change the estimates of migration (Table H of S1 File).

**Fig 7:**
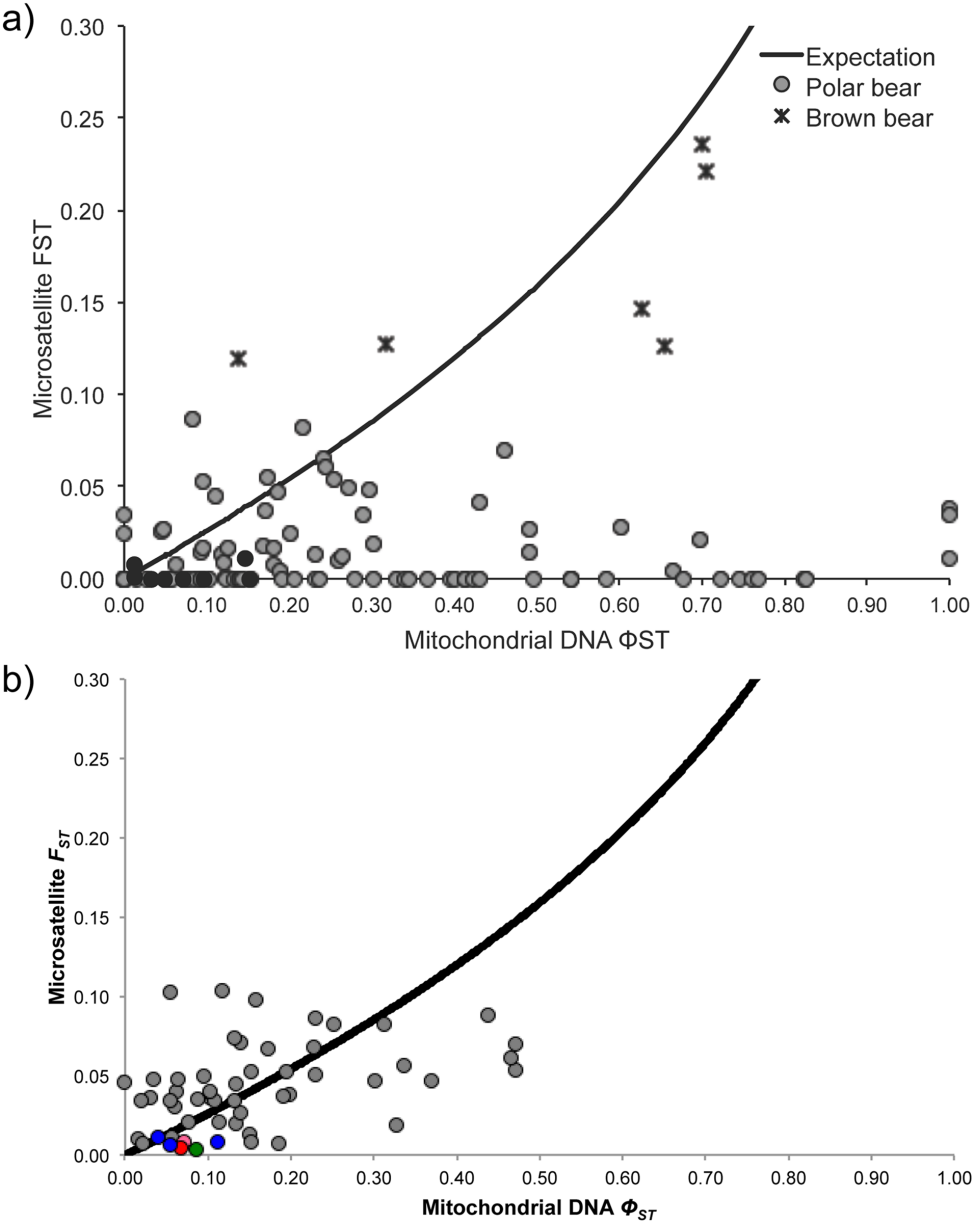
Comparisons of pairwise *F*_*ST*_ values for nuclear microsatellites with *Φ*_*ST*_ values from mitochondrial DNA; the line indicates the expectation of $F_{ST}(nu)=1-e^{0.25\times \text{ln}[1-F_{ST}(mt)]}$ under isolation as given in [56]. (a) S4 Fig from Peacock *et al*., 2015 [20], reproduced here under the terms of its Creative Commons CC0 license. (b) A recreated version of this graph, generated using our recalculated *F*_*ST*_ and *Φ*_*ST*_ values. In (b), M’Clintock Channel, Norwegian Bay, and Viscount Melville Sound were excluded because of inadequate sample sizes for mitochondrial DNA (*N* ≤ 3) and the Laptev Sea was excluded because this MU was significantly out of Hardy–Weinberg equilibrium. Points for brown bears were not recalculated and are not shown. Coloured points indicate intra-cluster MU pairs (coloured as in Fig 4); grey points indicate inter-cluster MU pairs.

To test for sex-biased dispersal across MU boundaries, we plotted pairwise *F*_*ST*_ for microsatellites against pairwise *Φ*_*ST*_ for mtDNA, as in Peacock *et al*., 2015 [20] (Fig 7b). In contrast to estimates from Peacock *et al*., 2015 [20], more points lie on or above the line of expectation (i.e., the line at which microsatellites differentiate populations as well as mitochondrial haplotypes), and the extreme values most supportive of strong male-biased dispersal, such as zero-estimates for microsatellites *F*_*ST*_ and one-estimates for mtDNA *Φ*_*ST*_ are absent. Inferences of sex-biased dispersal can also be made from the *R*-ratios of mitochondrial and nuclear *F*-statistics from AMOVAs, where *R*≪1 suggests female-biased dispersal and *R*≫4 suggests male-biased dispersal [80]. In our AMOVAs, *R*-ratios were *Φ*_*SC*_:*F*_*SC*_ = 8.3:1 for genetic variance among MUs within clusters and *Φ*_*CT*_:*F*_*CT*_ = 2.4:1 for genetic variance among clusters.

## Discussion

### Worldwide population structure of polar bears

In contrast to Peacock *et al*., 2015 [20], but similarly to Paetkau *et al*., 1999 [4], we detected four major genetic clusters of polar bears worldwide, additionally finding east–west sub-clusters in the Polar Basin and in the Canadian Archipelago. These findings corroborate previous studies of polar bear genetic structure [4, 12, 17, 19]. We note that we failed to detect a unique genetic cluster of bears on Akimiski Island in James Bay (Southern Hudson Bay) [10, 14, 20], which were not considered separately in this range-wide analysis because of low sample size. Our pairwise *F*_*ST*_ values between MUs were very similar to those calculated by Paetkau *et al*. 1999 [4], and differ tremendously from those in Peacock *et al*., 2015 [20], which appear to have been incorrectly calculated: most values in Peacock *et al*., 2015 [20] are negative, and they range as low as –0.26. Although negative values from the Weir–and–Cockerham estimator of *F*_*ST*_ [81] are possible (especially when sample sizes and sample variance in allele frequencies are low), they are typically not this extreme. We were unable to reproduce Peacock *et al*., 2015 [20]’s *F*_*ST*_ values using GenoDive, FSTAT, or Genepop on the full dataset; in all cases, the calculated values were similar to our own and those of Paetkau *et al*. 1999 [4]. Only when Arlequin was used under certain conditions were we able to reproduce these values. Specifically, the errant values in Peacock *et al*., 2015 [20] are an artefact caused by large amounts of missing data; they result only when one fails to enforce *any* missing-data cutoff in Arlequin (Table I of S1 File). When a reasonable missing data cutoff (e.g., 5%) is used, then sensible *F*_*ST*_ values consistent our own and those of Paetkau *et al*., 1999 [4] are produced (Table I of S1 File).

Under our grouping of MUs, the variance explained by genetic clusters (~4% for nuclear, ~9% for mitochondrial) was maximized through *K*-means clustering, and suggests moderate divergence among clusters. The four major genetic clusters are mostly separated by landmasses and multiyear ice that form barriers to gene flow for polar bears. The Hudson Complex and the Canadian Archipelago are separated by Baffin Island, Labrador, and the Melville Peninsula [3, 82]; the Archipelago and the Polar Basin are separated by Greenland in the east and by Banks and Victoria Islands in the west [3, 83]; and Norwegian Bay and the Archipelago are separated by thick multiyear ice, islands, and polynyas [3]. Genetic structure within the four major clusters is likely driven by broad-scale site fidelity to breeding and denning areas [3, 84, 85] and annual reuse of geographically predictable hunting grounds, such as tide cracks and lead systems [3, 86].

Based on our reanalysis of the original data from Peacock *et al*., 2015 [20], we have re-established Norwegian Bay as a distinct genetic cluster of polar bears near the northernmost reaches of Canada. Norwegian Bay is currently estimated to comprise 203 individuals (95% CI: 115–291; [87]), and—together with the neighbouring Queen Elizabeth Islands—it has previously been proposed as a separate designatable unit of polar bears based on ecological and genetic factors [88]. The status of this cluster is particularly relevant for polar bear conservation, as it is expected to coincide with Canada’s last sea-ice refugium [89]. This subpopulation has anecdotally been reported to be phenotypically unique [3], and we are currently conducting additional genetic analyses on this cluster, including genome scans on more recently collected samples. The Norwegian Bay cluster was likely not revealed in the analyses of Peacock *et al*., 2015 [20] because of highly unequal sample sizes, and perhaps also by the presence of many related individuals in Davis Strait, which can confound Structure analyses [25–27]. In addition, genetic clusters in Peacock *et al*., 2015 [20] were selected partially based on comparison of AMOVAs, and the existence of Norwegian Bay as a separate genetic cluster was not among the hypotheses tested (S7 Table of Peacock *et al*., 2015 [20]). In addition, all AMOVAs for microsatellites in Peacock *et al*., 2015 [20] have negative *Θ*_*SC*_ values or purportedly explain negative percentages of variance. We were unable to reproduce these unusual AMOVA results using Arlequin on the full dataset (e.g., Table J of S1 File).

Although an analysis of sex-biased dispersal was presented in Peacock *et al*., 2015 [20], it gave erroneous results because of incorrectly calculated *F*_*ST*_ values and the inclusion of populations with inadequate mtDNA sampling (Fig 7a). After correcting for these issues, we find there is little evidence that gene flow is strongly male-biased using the method in Peacock *et al*., 2015 [20]. In contrast, in AMOVAs, mitochondrial *Φ*_*SC*_ values were 8.3× nuclear *F*_*SC*_ values (whereas *Φ*_*CT*_ is only 2.4× *F*_*CT*_), which may suggest male-biased dispersal within—but not among—genetic clusters (however, cf. Fig 7b). Unfortunately, this comparison is hindered by different sampling regimes for mtDNA vs. nuclear DNA, including low within-cluster sampling of mtDNA (Table 2). In addition, any direct comparison of differentiation between uniparentally and diparentally inherited markers must be interpreted with caution, as such methods generally assume an effective-population-size ratio of 4:1, which is often untrue [90]. Though it would be better to perform sex-specific comparisons using nuclear markers, these methods may be underpowered unless bias in gene flow is extreme (i.e., 80:20), and they may also suffer from pseudoreplication [91, 92]. Therefore, the true extent of sex-biased dispersal in polar bears remains undetermined. Previous genetic studies have also reported conflicting findings of male-biased dispersal [19, 93], as have radio-telemetry studies of home-range size [94, 95]. Based on distances between recaptures, male polar bears appear to have only slightly larger home ranges than females, and this is perhaps because females move less when accompanied by cubs [3].

### Are polar bears migrating en masse into the Canadian Archipelago?

Polar bears rely on sea ice as a platform for locomotion [96], hunting [97], mating [98], and—in some areas—denning [99]. If climate change continues to reduce the extent and duration of Arctic sea ice, polar bears are likely to respond with altered movement patterns, resulting in increased mixing and gene flow between adjacent subpopulations [100]. To determine if changes in movement were already occurring, Peacock *et al*., 2015 [20] compared recent gene flow (i.e., over the past two generations) calculated using BayesAss with historical gene flow (i.e., time since the most recent common ancestor) calculated using Migrate [101]. They found an apparent reversal of gene flow over time, suggesting a recent influx of polar bears into the Canadian Archipelago from Southern Canada. However, the sampling regime for their BayesAss analysis was not described in the manuscript, and their results show known signs of non-convergence [21, 22]. A correction to the Supplementary Material of Peacock *et al*., 2015 [20] [102] published while our manuscript was in review states that individuals were randomly sampled from within the four populations used, with sample sizes of 26, 34, 60, and 60, for the Western Basin, Eastern Basin, Canadian Archipelago and Southern Canada, respectively. Unfortunately, BayesAss typically works best when sample sizes are much larger than this [21], and we were unable to reproduce these results using our own geographically balanced sampling regime with >100 samples per region.

In fact, within the Polar Basin, our BayesAss results detected exactly the opposite pattern of Peacock *et al*., 2015 [20]: namely, ~60-fold directional gene flow into the Eastern Polar Basin from the Western Polar Basin. This pattern held true in all 40 runs that included an Eastern–Western Polar Basin split. Similarly, our BayesAss results showed ~30-fold directional gene flow from the Western Archipelago into the Eastern Archipelago, though this pattern only held true in 8/20 runs; the remaining 12/20 runs suggested ~30-fold directional gene flow from the Eastern Archipelago into the Western Archipelago. Estimates of these immigration rates were close to the upper bound of 1/3 and—taken together—this suggests that *all estimates of gene flow within the Archipelago and the Polar Basin in both this paper and in Peacock et al., 2015 [20] are untrustworthy*, probably because of low genetic differentiation (*F*_*ST*_ ≈ 0.01) between these regions [21–23]. We similarly failed to confirm directional gene flow from the (Eastern) Polar Basin into the Canadian Archipelago; in all of our reanalyses, migration rates between these regions are not significantly directional. Although not significantly different, we did find that immigration into the Canadian Archipelago from Southern Canada (~4.9%) was slightly higher than in the reverse direction (~2.1%). The robustness of this finding across our different sampling regimes and the sampling regime of Peacock *et al*., 2015 [20] suggests that there may be slight northward gene flow into the Archipelago. Finally, we note that even our preferred BayesAss run (i.e., the 3-cluster run in Fig 6) may be interpreted as having not reached convergence, since proportions of non-migration have been estimated with small variance near the upper bound (Table E of S1 File). However, we believe that these estimates of low gene flow are realistic because the regions are largely separated by land and multiyear ice.

Among the first analyses conducted in Peacock *et al*., 2015 [20] were decadal comparisons of population structure to determine if it was safe to pool samples collected between the 1980s and the 2010s (S3 Table of Peacock *et al*., 2015 [20]). Their results showed that population composition had not changed significantly over this period in any of the regions examined. If polar bears had experienced substantial directional gene flow in response to recent climate change, it seems unlikely that this would not have resulted in detectable changes to population structure over this period, especially since Peacock *et al*., 2015 [20]’s high immigration rates of ~15% from both the Eastern Polar Basin and Southern Canada suggest that the Canadian Archipelago would likely not be demographically independent [103]. Although Arctic sea ice has been declining in thickness and extent in some regions since at least the 1950s [104, 105], the rapid loss of sea ice since the mid-1990s has been unprecedented over the last 1,450 years [106]. Therefore, we would expect to see changes in composition from the 1980s to the contemporary samples; however, no such changes were observed. Though our Structure plots suggest a substantial amount of migration and admixture among clusters, there is no clear pattern of directional gene flow. Further, these results might overestimate the amount of mating between genetic clusters, since Structure may be sensitive but not specific with respect to admixture [107], and cluster membership is estimated with extremely broad credible intervals when using a small number of markers [16, 108]. Therefore, we find the suggestion of mass gene flow into the Archipelago from Southern Canada and the Polar Basin uncompelling, and we strongly caution against managing Arctic Archipelago MUs as if they were being replenished by immigration.

## Conclusions

The three–four major genetic clusters selected in Peacock *et al*., 2015 [20] were selected based on faulty analyses, including miscalculated *F*_*ST*_ values, AMOVAs, and significance levels. The study was also compromised by highly unbalanced sample sizes and possibly by the inclusion of first- and second-degree relatives, as well as retention of large amounts of systematically missing data. One consequence of these data and analysis issues was the failure to detect a distinct subpopulation of polar bears in Norwegian Bay near Canada’s expected last sea-ice refugium. BayesAss results suggesting a recent influx of bears into the Archipelago and the Western Polar Basin showed known signs of non-convergence, and they were not supported in our own runs of the program. We therefore find the suggestion of strong recent directional gene flow into the Archipelago uncompelling. Many of these problems became obvious only upon examining the paper’s supplementary material; the original authors of Peacock *et al*., 2015 [20] should be commended for the well-documented results they made available, which allowed us to detect the issues in their study. Recently, supplementary material has been accused of being poorly peer-reviewed, thereby threatening the integrity of the scientific literature [109]. We hope that this example will serve as a reminder to both authors and reviewers to scrutinize this supplementary material more closely in the future. In the interest of even greater openness, we have deposited inputs, outputs, and scripts used to perform our analyses at Open Science Framework (http://osf.io/kqcr4). We encourage both reviewers and readers to further explore this invaluable dataset.

## Supporting Information

S1 File Complete set of supporting information figures and tables. (DOCX)
