# Supplementary material for: Circumpolar Genetic Structure and Recent Gene Flow of Polar Bears: A Reanalysis
Source: PLoS One. 2016 Mar 14;11(3):e0148967. doi: 10.1371/journal.pone.0148967 (PMC4790856; doi:10.1371/journal.pone.0148967)
Supplement: S1 File — (DOCX) [file pone.0148967.s001.docx]

**Supplementary Material**

**Table A.** Individuals retained for all main analyses in this paper (except for BayesAss estimates of migration rates). For a small number of individuals, there were discrepancies in the subpopulation designations between the list provided in Table S11 of Peacock *et al.*, 2015 and the listed provided in the microsatellite dataset on Dryad (doi:10.5061/dryad.v2j1r). These were assumed to represent individuals who were sampled multiple times across population boundaries (i.e., temporary or permanent migrants) or individuals whose subpopulation designations were changed according to Supporting Information S1 of Peacock *et al.*, 2015. Where these discrepancies existed, we have used the populations of origin from the microsatellite dataset. These are indicated by the first two characters in each individual ID, and correspond to the abbreviations in Table 1.

| **ID** | **ID** | **ID** | **ID** | **ID** | **ID** | **ID** | **ID** | **ID** |
| --- | --- | --- | --- | --- | --- | --- | --- | --- |
| BB14216 | BS7922 | DS18893 | FB28223 | KB13558 | LS13690 | NB10461 | SB20424 | VM13057 |
| BB14411 | BS7926 | DS30620 | FB28234 | KB13560 | LS13713 | NB10468 | SB20457 | VM13061 |
| BB14436 | BS7935 | DS30814 | FB28249 | KB13561 | LS14154 | NB10744 | SB20580 | VM13067 |
| BB14459 | BS7997 | DS35017 | FB28252 | KB13564 | LS14166 | NB12020 | SB20668 | VM13130 |
| BB14801 | BS98398 | DS35208 | FB28253 | KB13720 | LS14175 | NB12021 | SB20735 | VM13133 |
| BB18293 | CS121 | DS35235 | FB28254 | KB13792 | LS14178 | NB12026 | SB20764 | VM13137 |
| BB18299 | CS138 | DS35237 | FB28258 | KB13795 | LS14334 | NB12027 | SB20886 | VM13140 |
| BB18305 | CS139 | DS35514 | FB28261 | KB13796 | LS14503 | NB12030 | SB20987 | VM13141 |
| BB18327 | CS20688 | DS35711 | FB28266 | KB14488 | LS25670 | NB12031 | SB20988 | VM13144 |
| BB18333 | CS21058 | DS35774 | FB28270 | KB14581 | LS28036 | NB12034 | SB20990 | VM13148 |
| BB18348 | CS21098 | EG14416 | FB28276 | KB14582 | LS28041 | NB12035 | SB21002 | VM13151 |
| BB23848 | CS21106 | EG14417 | FB30602 | KB14583 | LS28056 | NB12036 | SB21219 | VM13154 |
| BB23864 | CS21120 | EG14418 | FB30606 | KB14591 | LS28073 | NB12038 | SB21221 | VM13155 |
| BB23870 | CS21137 | EG14419 | FB35163 | KB14592 | LS28078 | NB12041 | SB2525 | VM13158 |
| BB23871 | CS21160 | EG14420 | FB35800 | KB14607 | LS28079 | NB12042 | SB32260 | VM13242 |
| BB23872 | CS21170 | EG14421 | GB13674 | KB14616 | LS28086 | NW04142 | SB32267 | VM13245 |
| BB23875 | CS21182 | EG14422 | GB13675 | KB14617 | LS28111 | NW13340 | SB6020 | VM13249 |
| BB35026 | CS21183 | EG14423 | GB13678 | KB14626 | LS28112 | NW13344 | SB6098 | VM13251 |
| BB35076 | CS21491 | EG14425 | GB14261 | KB14627 | LS28115 | NW13345 | SB6538 | VM13252 |
| BB35079 | CS21494 | EG14426 | GB14262 | KB15269 | LS28117 | NW13351 | SB6545 | VM13255 |
| BB35082 | CS21496 | EG14427 | GB14364 | KS20035 | LS28120 | NW13502 | SH16834 | VM13257 |
| BB35092 | CS21497 | EG14428 | GB18125 | KS20036 | LS28121 | NW13700 | SH16866 | VM13259 |
| BB35098 | CS21503 | EG14429 | GB19235 | KS20037 | LS28122 | NW13701 | SH16868 | VM13272 |
| BB35641 | CS21508 | EG14430 | GB20821 | KS20038 | LS28124 | NW13704 | SH16888 | VM13274 |
| BB35646 | CS21512 | EG14431 | GB20864 | KS20043 | LS28125 | NW13716 | SH16912 | VM13276 |
| BB35649 | CS21521 | EG14432 | GB20866 | KS20050 | LS29150 | NW13719 | SH16914 | WH00563 |
| BB35652 | CS57 | EG7119 | GB21562 | KS20051 | MC13465 | NW14024 | SH16916 | WH04151 |
| BB35664 | CS6575 | EG7120 | GB21567 | KS20052 | MC13466 | NW14029 | SH16918 | WH04198 |
| BB35739 | CS6738 | EG7121 | GB21615 | KS20056 | MC13666 | NW14515 | SH16920 | WH05990 |
| BB35745 | CS6865 | EG7123 | GB21617 | KS20057 | MC13668 | NW14516 | SH16925 | WH10565 |
| BS23016 | CS6875 | EG7124 | GB27786 | KS20060 | MC14975 | NW14517 | SH16940 | WH10575 |
| BS23060 | CS6881 | EG7125 | GB27875 | KS20061 | MC14976 | NW14519 | SH16983 | WH10602 |
| BS23174 | CS6950 | EG7127 | GB27882 | KS7980 | MC14977 | NW14521 | SH16987 | WH10614 |
| BS23177 | CS6974 | EG7128 | GB27923 | KS7982 | MC14978 | NW14522 | SH30576 | WH10631 |
| BS23294 | CS6980 | EG7129 | GB27924 | KS7984 | MC14979 | NW14523 | SH30889 | WH10650 |
| BS23357 | DS18043 | EG7131 | GB27926 | KS7986 | MC21393 | NW14524 | SH30936 | WH10651 |
| BS23441 | DS18069 | EG7132 | GB27927 | KS7988 | MC21396 | NW14529 | SH37004 | WH11134 |
| BS23460 | DS18141 | EG7133 | GB27963 | LP105 | MC21551 | NW14530 | SH37009 | WH11138 |
| BS23479 | DS18256 | EG7351 | GB27964 | LP129 | MC21927 | NW14536 | SH37010 | WH11345 |
| BS23497 | DS18279 | EG7352 | GB27966 | LP130 | MC21928 | NW14537 | SH37012 | WH12380 |
| BS23513 | DS18292 | FB17541 | GB27976 | LP132 | NB02573 | NW14540 | SH37013 | WH17447 |
| BS23538 | DS18320 | FB17545 | GB28023 | LP145 | NB03541 | NW14613 | SH37014 | WH19210 |
| BS23616 | DS18366 | FB18528 | GB28024 | LP20044 | NB09876 | NW15285 | SH37015 | WH19962 |
| BS23625 | DS18395 | FB18529 | GB28297 | LP20045 | NB10000 | NW15287 | SH37016 | WH25818 |
| BS23683 | DS18407 | FB18839 | GB28733 | LP20046 | NB10025 | NW15299 | SH37018 | WH25859 |
| BS23703 | DS18451 | FB18978 | KB13167 | LP20047 | NB10046 | SB06488 | SH37022 | WH27803 |
| BS23707 | DS18475 | FB18987 | KB13168 | LP6985 | NB10050 | SB06835 | SH37023 | WH27859 |
| BS23744 | DS18624 | FB18990 | KB13175 | LP6989 | NB10051 | SB09823 | SH37037 | WH27863 |
| BS23750 | DS18641 | FB18997 | KB13329 | LP6990 | NB10168 | SB09837 | SH37046 | WH27865 |
| BS23760 | DS18648 | FB22062 | KB13335 | LP6993 | NB10299 | SB09958 | SH37047 | WH27871 |
| BS23822 | DS18656 | FB22068 | KB13336 | LP6994 | NB10300 | SB09970 | VM02807 | WH27872 |
| BS23845 | DS18783 | FB22069 | KB13337 | LS03888 | NB10303 | SB10413 | VM08677 | WH27873 |
| BS7815 | DS18811 | FB28209 | KB13554 | LS08543 | NB10307 | SB20184 | VM08919 | WH28093 |
| BS7837 | DS18852 | FB28213 | KB13556 | LS13687 | NB10448 | SB20206 | VM08958 | WH28097 |
| BS7914 | DS18878 | FB28222 | KB13557 | LS13688 | NB10460 | SB20334 | VM13052 | WH28098 |

**Table B.** Significance of pairwise genic differentiation (below diagonal) and genotypic differentiation (above diagonal) as calculated for nuclear microsatellites in GenePop. Significant values after a Holm correction for the number of tests are indicated with “+”, non-significant tests are indicated with a “-”. For a description of boxes and shading, see the main manuscript. Values for the Laptev Sea are not shown as this MU was significantly out of Hardy–Weinberg equilibrium.

|  | **SH** | **WH** | **FB** | **DS** | **BB** | **KB** | **LS** | **GB** | **MC** | **VM** | **NW** | **NB** | **SB** | **CS** | **LP** | **KS** | **BS** | **EG** |
| --- | --- | --- | --- | --- | --- | --- | --- | --- | --- | --- | --- | --- | --- | --- | --- | --- | --- | --- |
| **SH** |  | - | + | + | + | + | + | + | + | + | + | + | + | + |  | + | + | + |
| **WH** | - |  | - | + | + | + | + | + | + | + | + | + | + | + |  | + | + | + |
| **FB** | + | - |  | - | + | + | + | + | + | + | + | + | + | + |  | + | + | + |
| **DS** | + | + | - |  | - | + | + | + | + | + | + | + | + | + |  | + | + | + |
| **BB** | + | + | + | - |  | - | + | + | + | + | + | + | + | + |  | + | + | + |
| **KB** | + | + | + | + | - |  | + | + | + | + | + | + | + | + |  | + | + | + |
| **LS** | + | + | + | + | + | + |  | - | - | - | + | + | + | + |  | + | + | + |
| **GB** | + | + | + | + | + | + | - |  | + | + | + | + | + | + |  | + | + | + |
| **MC** | + | + | + | + | + | + | - | + |  | - | + | + | + | + |  | + | + | + |
| **VM** | + | + | + | + | + | + | - | + | - |  | + | + | + | + |  | + | + | + |
| **NW** | + | + | + | + | + | + | + | + | + | + |  | + | + | + |  | + | + | + |
| **NB** | + | + | + | + | + | + | + | + | + | + | + |  | - | - |  | - | + | + |
| **SB** | + | + | + | + | + | + | + | + | + | + | + | - |  | - |  | - | + | + |
| **CS** | + | + | + | + | + | + | + | + | + | + | + | - | - |  |  | - | + | + |
| **LP** |  |  |  |  |  |  |  |  |  |  |  |  |  |  |  |  |  |  |
| **KS** | + | + | + | + | + | + | + | + | + | + | + | - | + | + |  |  | - | - |
| **BS** | + | + | + | + | + | + | + | + | + | + | + | + | + | + |  | - |  | - |
| **EG** | + | + | + | + | + | + | + | + | + | + | + | + | + | + |  | - | - |  |

**Table C.** Significance of exact test of population differentiation (below diagonal) and pairwise *F_ST_* (above diagonal) as calculated for mitochondrial DNA haplotypes in Arlequin. Significant values after a Holm correction for the number of tests are indicated with “+”, non-significant tests are indicated with a “-”. For a description of boxes and shading, see the main manuscript. Values for the Laptev Sea are not shown as this MU was significantly out of Hardy–Weinberg equilibrium. Values for M’Clintock Channel, Norwegian Bay, and Viscount Melville are not shown because sample sizes were inadequate to estimate haplotype frequencies (i.e., N≤3). All other rows/columns missing data were not genotyped for mitochondrial DNA.

|  | **SH** | **WH** | **FB** | **DS** | **BB** | **KB** | **LS** | **GB** | **MC** | **VM** | **NW** | **NB** | **SB** | **CS** | **LP** | **KS** | **BS** | **EG** |
| --- | --- | --- | --- | --- | --- | --- | --- | --- | --- | --- | --- | --- | --- | --- | --- | --- | --- | --- |
| **SH** |  | + | - | + | + |  | + | - |  |  |  |  | + | + |  | + | + |  |
| **WH** | + |  | + | + | + |  | + | + |  |  |  |  | + | + |  | + | + |  |
| **FB** | - | + |  | - | - |  | + | - |  |  |  |  | + | + |  | + | + |  |
| **DS** | + | + | - |  | + |  | + | - |  |  |  |  | + | + |  | + | + |  |
| **BB** | + | + | + | + |  |  | - | - |  |  |  |  | + | + |  | + | + |  |
| **KB** |  |  |  |  |  |  |  |  |  |  |  |  |  |  |  |  |  |  |
| **LS** | + | + | + | + | - |  |  | - |  |  |  |  | + | + |  | + | + |  |
| **GB** | - | + | - | - | - |  | - |  |  |  |  |  | + | + |  | + | + |  |
| **MC** |  |  |  |  |  |  |  |  |  |  |  |  |  |  |  |  |  |  |
| **VM** |  |  |  |  |  |  |  |  |  |  |  |  |  |  |  |  |  |  |
| **NW** |  |  |  |  |  |  |  |  |  |  |  |  |  |  |  |  |  |  |
| **NB** |  |  |  |  |  |  |  |  |  |  |  |  |  |  |  |  |  |  |
| **SB** | + | + | + | + | + |  | + | + |  |  |  |  |  | + |  | - | + |  |
| **CS** | - | + | + | + | + |  | + | - |  |  |  |  | + |  |  | + | + |  |
| **LP** |  |  |  |  |  |  |  |  |  |  |  |  |  |  |  |  |  |  |
| **KS** | + | + | + | + | + |  | + | + |  |  |  |  | - | - |  |  | - |  |
| **BS** | + | + | + | + | + |  | + | + |  |  |  |  | + | + |  | - |  |  |
| **EG** |  |  |  |  |  |  |  |  |  |  |  |  |  |  |  |  |  |  |

**Table D.** Sampling scheme used for BayesAss analyses. We attempted to obtain 150 samples per cluster (min. 100) such that each management unit (MU) was proportionally represented according to its population size. Population sizes are estimated with broad confidence intervals, and many estimated population sizes (indicated with question marks below) are rough guesses that have not been accepted by the IUCN Polar Bear Specialist Group. Individuals were selected blindly (i.e., without viewing their cluster membership) while trying to obtain geographically representative sampling within each MU. We note that although Foxe Basin makes up approximately 50% of the Hudson cluster, sampling in the northern part of this MU (i.e., Foxe Basin *sensu stricto*) is poor in the original dataset. Therefore, we used additional samples from the Hudson Strait portion of this MU in their place. Polar bears from Hudson Strait appear to be genetically similar to northern Foxe Basin (and southern Davis Strait) [1].

|  |  | **3 out of 4 clusters** | | | | | **4 out of 5 clusters (PC2015)** | | | | | **5 out of 6 clusters** | | | | |
| --- | --- | --- | --- | --- | --- | --- | --- | --- | --- | --- | --- | --- | --- | --- | --- | --- |
| **MU** | **Population size** | **Cluster** | **Proportion of cluster** | **Samples to use** | **Actually used** | **Mean YoC** | **Cluster** | **Proportion of cluster** | **Samples to use** | **Actually used** | **Mean YoC** | **Cluster** | **Proportion of cluster** | **Samples to use** | **Actually used** | **Mean YoC** |
| **WH** | 1030 [2] | Hudson | 0.20 | 30 | 30 | 1996 | Hudson | 0.20 | 30 | 30 | 1996 | Hudson | 0.20 | 30 | 30 | 1996 |
| **SH** | 951 [2] | Hudson | 0.19 | 28 | 28 | 2008 | Hudson | 0.19 | 28 | 28 | 2008 | Hudson | 0.19 | 28 | 28 | 2008 |
| **FB** | 2580 [2] | Hudson | 0.50 | 76 | 76 | 1999 | Hudson | 0.50 | 76 | 76 | 1999 | Hudson | 0.50 | 76 | 76 | 1999 |
| **sDS** | 557 [3] | Hudson | 0.11 | 16 | 16 | 2004 | Hudson | 0.11 | 16 | 16 | 2004 | Hudson | 0.11 | 16 | 16 | 2004 |
| **nDS** | 1602 [3] | Archipelago | 0.20 | 30 | 30 | 2007 | Archipelago | 0.20 | 30 | 30 | 2007 | E. Archipelago | 0.48 | 73 | 73 | 2005 |
| **BB** | 1546 [2] | Archipelago | 0.20 | 29 | 29 | 1998 | Archipelago | 0.20 | 29 | 29 | 1998 | E. Archipelago | 0.47 | 70 | 70 | 2000 |
| **KB** | 164 [2] | Archipelago | 0.02 | 3 | 3 | 1995 | Archipelago | 0.02 | 3 | 3 | 1995 | E. Archipelago | 0.05 | 7 | 7 | 1994 |
| **LS** | 2541 [2] | Archipelago | 0.32 | 48 | 48 | 2001 | Archipelago | 0.32 | 48 | 48 | 2001 | W. Archipelago | 0.56 | 83 | 65 | 2001 |
| **MC** | 284 [2] | Archipelago | 0.04 | 5 | 5 | 1996 | Archipelago | 0.04 | 5 | 5 | 1996 | W. Archipelago | 0.06 | 9 | 7 | 1996 |
| **GB** | 1592 [2] | Archipelago | 0.20 | 30 | 30 | 2000 | Archipelago | 0.20 | 30 | 30 | 2000 | W. Archipelago | 0.35 | 52 | 37 | 2000 |
| **VM** | 161 [2] | Archipelago | 0.02 | 3 | 3 | 1992 | Archipelago | 0.02 | 3 | 3 | 1992 | W. Archipelago | 0.04 | 5 | 4 | 1992 |
| **NB** | 980 [2] | Basin | 0.06 | 10 | 10 | 1989 | W. Basin | 0.17 | 25 | 25 | 1989 | W. Basin | 0.17 | 25 | 25 | 1989 |
| **SB** | 907 [2] | Basin | 0.06 | 9 | 9 | 1994 | W. Basin | 0.15 | 23 | 23 | 1996 | W. Basin | 0.15 | 23 | 23 | 1996 |
| **CS** | 3500? [2, 4] | Basin | 0.23 | 34 | 34 | 1991 | W. Basin | 0.59 | 89 | 89 | 1990 | W. Basin | 0.59 | 89 | 89 | 1990 |
| **wLP** | 500? [2, 4] | Basin | 0.03 | 5 | 5 | 2004 | W. Basin | 0.08 | 13 | 9 | 2001 | W. Basin | 0.08 | 13 | 9 | 2001 |
| **eLP** | 500? [2, 4] | Basin | 0.03 | 5 | 5 | 1994 | E. Basin | 0.05 | 8 | 4 | 1994 | E. Basin | 0.05 | 8 | 4 | 1994 |
| **KS** | 3200? [5] | Basin | 0.21 | 32 | 17 | 1994 | E. Basin | 0.34 | 51 | 17 | 1994 | E. Basin | 0.34 | 51 | 17 | 1994 |
| **BS** | 2644 [2] | Basin | 0.17 | 26 | 26 | 2001 | E. Basin | 0.28 | 42 | 49 | 2001 | E. Basin | 0.28 | 42 | 49 | 2001 |
| **EG** | 3000? [4] | Basin | 0.20 | 30 | 30 | 1990 | E. Basin | 0.32 | 48 | 30 | 1990 | E. Basin | 0.32 | 48 | 30 | 1990 |
|  | **Missing data** | **14-locus dataset: 0%; 21-locus dataset: 11.8%** | | | | | **14-locus dataset: 0.03%; 21-locus dataset: 11.4%** | | | | | **14-locus dataset: 0.02%; 21-locus dataset: 11.7%** | | | | |

**Table E.** Proportions of migrant and non-migrant ancestry from BayesAss for *K*=4 (minus Norwegian Bay). Each cell indicates the per-generation fraction of individuals from the population named in that *row* who are migrants from the population named in that *column*. Diagonals indicate the proportion of non-migrants. Numbers in parentheses indicate standard errors. Settings used to obtain good mixing (i.e., acceptance ratios of 0.2–0.6) were: Δ*_A_*=0.15, Δ*_M_*=0.1, Δ*_F_*=0.2.

|  | **Archipelago** | **Polar Basin** | **Hudson Complex** |
| --- | --- | --- | --- |
| **Archipelago** | 0.9394 (0.0190) | 0.0114 (0.0096) | 0.0492 (0.0164) |
| **Polar Basin** | 0.0184 (0.0128) | 0.9744 (0.0134) | 0.0072 (0.0055) |
| **Hudson Complex** | 0.0211 (0.0102) | 0.0088 (0.0060) | 0.9701 (0.0113) |

**Table F.** Proportions of migrant and non-migrant ancestry from BayesAss for *K*=5 (minus Norwegian Bay). This population grouping corresponds roughly to the four-population grouping presented in PC2015, with the exception that Norwegian Bay has been removed from the Archipelago, and the Eastern/Western Polar Basin have been split at the large sampling discontinuity in the Laptev Sea. Each cell indicates the per-generation fraction of individuals from the population named in that *row* who are migrants from the population named in that *column*. Diagonals indicate the proportion of non-migrants. Numbers in parentheses indicate standard errors. Settings used to obtain good mixing (i.e., acceptance ratios of 0.2–0.6) were: Δ*_A_*=0.15, Δ*_M_*=0.1, Δ*_F_*=0.25

|  | **Archipelago** | **Eastern Basin** | **Hudson Complex** | **Western Basin** |
| --- | --- | --- | --- | --- |
| **Archipelago** | 0.9350 (0.0183) | 0.0046 (0.0043) | 0.0500 (0.0161) | 0.0104 (0.0082) |
| **Eastern Basin** | 0.0095 (0.0083) | 0.6734 (0.0074) | 0.0048 (0.0045) | 0.3123 (0.0123) |
| **Hudson Complex** | 0.0207 (0.0101) | 0.0040 (0.0038) | 0.9673 (0.0113) | 0.0080 (0.0056) |
| **Western Basin** | 0.0064 (0.0056) | 0.0055 (0.0049) | 0.0027 (0.0026) | 0.9855 (0.0077) |

**Table G.** Proportions of migrant and non-migrant ancestry from BayesAss for *K*=6 (minus Norwegian Bay). Each cell indicates the per-generation fraction of individuals from the population named in that *row* who are migrants from the population named in that *column*. Diagonals indicate the proportion of non-migrants. Numbers in parentheses indicate standard errors. Settings used to obtain good mixing (i.e., acceptance ratios of 0.2–0.6) were: Δ*_A_*=0.2, Δ*_M_*=0.1, Δ*_F_*=0.25.

|  | **Eastern Archipelago** | **Eastern Basin** | **Hudson Complex** | **Western Archipelago** | **Western Basin** |
| --- | --- | --- | --- | --- | --- |
| **Eastern Archipelago** | 0.6770 (0.0100) | 0.0032 (0.0031) | 0.0568 (0.0199) | 0.2581 (0.00216) | 0.0049 (0.0042) |
| **Eastern Basin** | 0.0047 (0.0046) | 0.6738 (0.0081) | 0.0050 (0.0047) | 0.0102 (0.0087) | 0.3063 (0.0136) |
| **Hudson Complex** | 0.0281 (0.0131) | 0.0037 (0.0035) | 0.9529 (0.0145) | 0.0081 (0.0063) | 0.0071 (0.0050) |
| **Western Archipelago** | 0.0106 (0.0081) | 0.0054 (0.0051) | 0.0134 (0.0095) | 0.9554 (0.0156) | 0.0153 (0.0102) |
| **Western Basin** | 0.0038 (0.0035) | 0.0052 (0.0047) | 0.0027 (0.0027) | 0.0055 (0.0050) | 0.9829 (0.0079) |

**Table H.** Proportions of migrant and non-migrant ancestry from BayesAss for *K*=5 (minus Norwegian Bay) with the additional removal of all samples from the Laptev Sea. Each cell indicates the per-generation fraction of individuals from the population named in that *row* who are migrants from the population named in that *column*. Diagonals indicate the proportion of non-migrants. Numbers in parentheses indicate standard errors. Settings used to obtain good mixing (i.e., acceptance ratios of 0.2–0.6) were: Δ*_A_*=0.15, Δ*_M_*=0.1, Δ*_F_*=0.25.

|  | **Archipelago** | **Eastern Basin** | **Hudson Complex** | **Western Basin** |
| --- | --- | --- | --- | --- |
| **Archipelago** | 0.9354 (0.0189) | 0.0048 (0.0045) | 0.0496 (0.0165) | 0.0102 (0.0082) |
| **Eastern Basin** | 0.0102 (0.0088) | 0.6726 (0.0062) | 0.0056 (0.0053) | 0.3117 (0.0119) |
| **Hudson Complex** | 0.0207 (0.0102) | 0.0039 (0.0037) | 0.9672 (0.0116) | 0.0082 (0.0057) |
| **Western Basin** | 0.0073 (0.0062) | 0.0054 (0.0048) | 0.0029 (0.0029) | 0.9845 (0.0082) |

**Table I.** Pairwise *F_ST_* values for nuclear microsatellites recalculated in Arlequin using the complete dataset from PC2015. Values above the diagonal were calculated using a missing data cutoff of 0.05, which resulted in the exclusion of the same seven loci described in the main manuscript. Values below the diagonal were calculated using a missing data cutoff of 1 (i.e., including all 21 loci), which results in many highly negative *F_ST_* values. Values below the diagonal are identical to those presented in Table S5 of PC2015, except that they are shifted up by one. For instance, in PC2015, –0.113 corresponds to the *F_ST_* for BB and CS, rather than for BB and BS. This suggests that—in addition to miscalculation of *F_ST_* caused by large amounts of missing data—the *F_ST_* values in PC2015 are also incorrect due to copy–paste error.

|  | **BB** | **BS** | **CS** | **DS** | **EG** | **FB** | **GB** | **KB** | **KS** | **LP** | **LS** | **MC** | **NB** | **NW** | **SB** | **SH** | **VM** | **WH** |
| --- | --- | --- | --- | --- | --- | --- | --- | --- | --- | --- | --- | --- | --- | --- | --- | --- | --- | --- |
| **BB** |  | 0.031 | 0.042 | 0.007 | 0.028 | 0.019 | 0.017 | 0.004 | 0.031 | 0.045 | 0.009 | 0.015 | 0.033 | 0.031 | 0.039 | 0.036 | 0.026 | 0.043 |
| **BS** | –0.113 |  | 0.017 | 0.039 | 0.004 | 0.056 | 0.047 | 0.034 | 0.002 | 0.017 | 0.037 | 0.027 | 0.016 | 0.057 | 0.017 | 0.083 | 0.036 | 0.076 |
| **CS** | 0.018 | –0.177 |  | 0.052 | 0.022 | 0.072 | 0.051 | 0.052 | 0.009 | 0.006 | 0.046 | 0.033 | 0.004 | 0.068 | 0.004 | 0.099 | 0.043 | 0.092 |
| **DS** | 0.004 | –0.113 | 0.027 |  | 0.039 | 0.005 | 0.025 | 0.019 | 0.038 | 0.058 | 0.020 | 0.026 | 0.042 | 0.040 | 0.046 | 0.023 | 0.034 | 0.025 |
| **EG** | –0.058 | –0.126 | –0.124 | –0.075 |  | 0.055 | 0.045 | 0.027 | 0.008 | 0.018 | 0.031 | 0.026 | 0.016 | 0.053 | 0.021 | 0.086 | 0.039 | 0.081 |
| **FB** | 0.017 | –0.080 | 0.041 | 0.001 | –0.023 |  | 0.034 | 0.030 | 0.054 | 0.079 | 0.030 | 0.039 | 0.059 | 0.049 | 0.062 | 0.011 | 0.045 | 0.010 |
| **GB** | –0.013 | –0.064 | –0.004 | –0.022 | 0.020 | 0.008 |  | 0.024 | 0.050 | 0.057 | 0.012 | 0.013 | 0.039 | 0.046 | 0.046 | 0.044 | 0.026 | 0.051 |
| **KB** | –0.086 | –0.073 | –0.087 | –0.101 | 0.030 | –0.054 | 0.003 |  | 0.038 | 0.056 | 0.008 | 0.014 | 0.035 | 0.026 | 0.047 | 0.050 | 0.023 | 0.052 |
| **KS** | 0.010 | –0.263 | 0.008 | 0.014 | –0.161 | 0.027 | –0.005 | –0.112 |  | 0.012 | 0.037 | 0.022 | 0.010 | 0.065 | 0.009 | 0.083 | 0.032 | 0.075 |
| **LP** | 0.030 | –0.190 | 0.003 | 0.038 | –0.107 | 0.059 | 0.016 | –0.055 | 0.004 |  | 0.056 | 0.050 | 0.019 | 0.079 | 0.010 | 0.112 | 0.059 | 0.101 |
| **LS** | –0.010 | –0.081 | 0.020 | –0.012 | –0.014 | 0.012 | 0.011 | –0.039 | 0.017 | 0.042 |  | –0.001 | 0.030 | 0.026 | 0.043 | 0.049 | 0.010 | 0.049 |
| **MC** | –0.077 | –0.078 | –0.106 | –0.098 | 0.029 | –0.049 | –0.007 | 0.012 | –0.130 | –0.065 | –0.050 |  | 0.025 | 0.043 | 0.033 | 0.061 | 0.010 | 0.057 |
| **NB** | –0.054 | –0.111 | –0.142 | –0.072 | 0.014 | –0.020 | 0.015 | 0.038 | –0.157 | –0.107 | –0.016 | 0.028 |  | 0.050 | 0.005 | 0.086 | 0.026 | 0.080 |
| **NW** | –0.060 | –0.042 | –0.071 | –0.081 | 0.055 | –0.036 | 0.024 | 0.023 | –0.086 | –0.032 | –0.024 | 0.038 | 0.051 |  | 0.062 | 0.067 | 0.036 | 0.066 |
| **SB** | 0.045 | –0.135 | –0.025 | 0.049 | –0.064 | 0.066 | 0.013 | –0.034 | –0.021 | –0.005 | 0.026 | –0.048 | –0.080 | –0.020 |  | 0.086 | 0.038 | 0.079 |
| **SH** | 0.000 | –0.146 | 0.087 | –0.008 | –0.081 | –0.028 | –0.018 | –0.119 | 0.070 | 0.082 | 0.013 | –0.113 | –0.082 | –0.105 | 0.056 |  | 0.064 | 0.009 |
| **VM** | –0.063 | –0.077 | –0.100 | –0.083 | 0.038 | –0.037 | 0.004 | 0.025 | –0.130 | –0.062 | –0.037 | 0.011 | 0.026 | 0.035 | –0.047 | –0.108 |  | 0.059 |
| **WH** | 0.018 | –0.027 | 0.051 | –0.013 | 0.052 | –0.010 | 0.053 | 0.021 | 0.042 | 0.076 | 0.050 | 0.021 | 0.049 | 0.035 | 0.061 | –0.042 | 0.028 |  |

**Table J.** Examples of locus-by-locus AMOVAs calculated in Arlequin using a 5% missing data cutoff for 21 microsatellites. Significance was determined using 1000 permutations. Note that many potential population groupings such as the ones below were discounted in PC2015 because of apparently incorrectly calculated AMOVAs, which often included negative percentage variance explained for *F_SC_*. We were unable to replicate these results; rather, all *F*-values are highly significant.

| **Hypothesis** | **Source of variation** | **% variance** | ***F*-statistic** | ***F*-value** | ***P*-value** |
| --- | --- | --- | --- | --- | --- |
| Proposed Canadian Conservation Units  (Hypothesis D of PC2015, Table S7) | Within individuals | 96.04% | *F_IT_* | 0.040 | 0 |
|  | Among individuals in MUs | 0.84% | *F_IS_* | 0.009 | 0.001 |
|  | Among MUs in clusters | 1.18% | *F_SC_* | 0.012 | 0 |
|  | Among clusters | 1.95% | *F_CT_* | 0.019 | 0 |
| Proposed Canadian Conservation Units  (including a separate cluster for Norwegian Bay) | Within individuals | 95.96% | *F_IT_* | 0.040 | 0 |
|  | Among individuals in MUs | 0.86% | *F_IS_* | 0.009 | 0.002 |
|  | Among MUs in clusters | 1.18% | *F_SC_* | 0.012 | 0 |
|  | Among clusters | 2.00% | *F_CT_* | 0.020 | 0 |


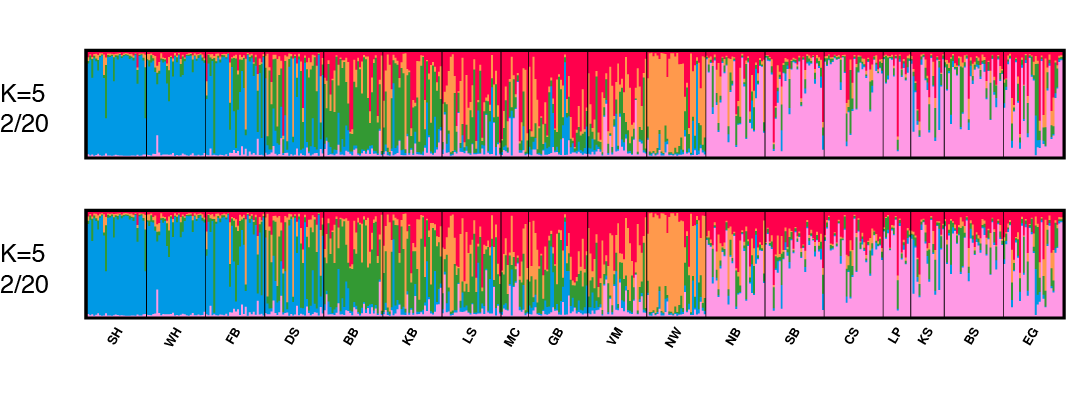


**Figure A.** CLUMPAK-averaged minority modes for *K*=5. The majority mode is shown in Fig 2 of the main paper.

**
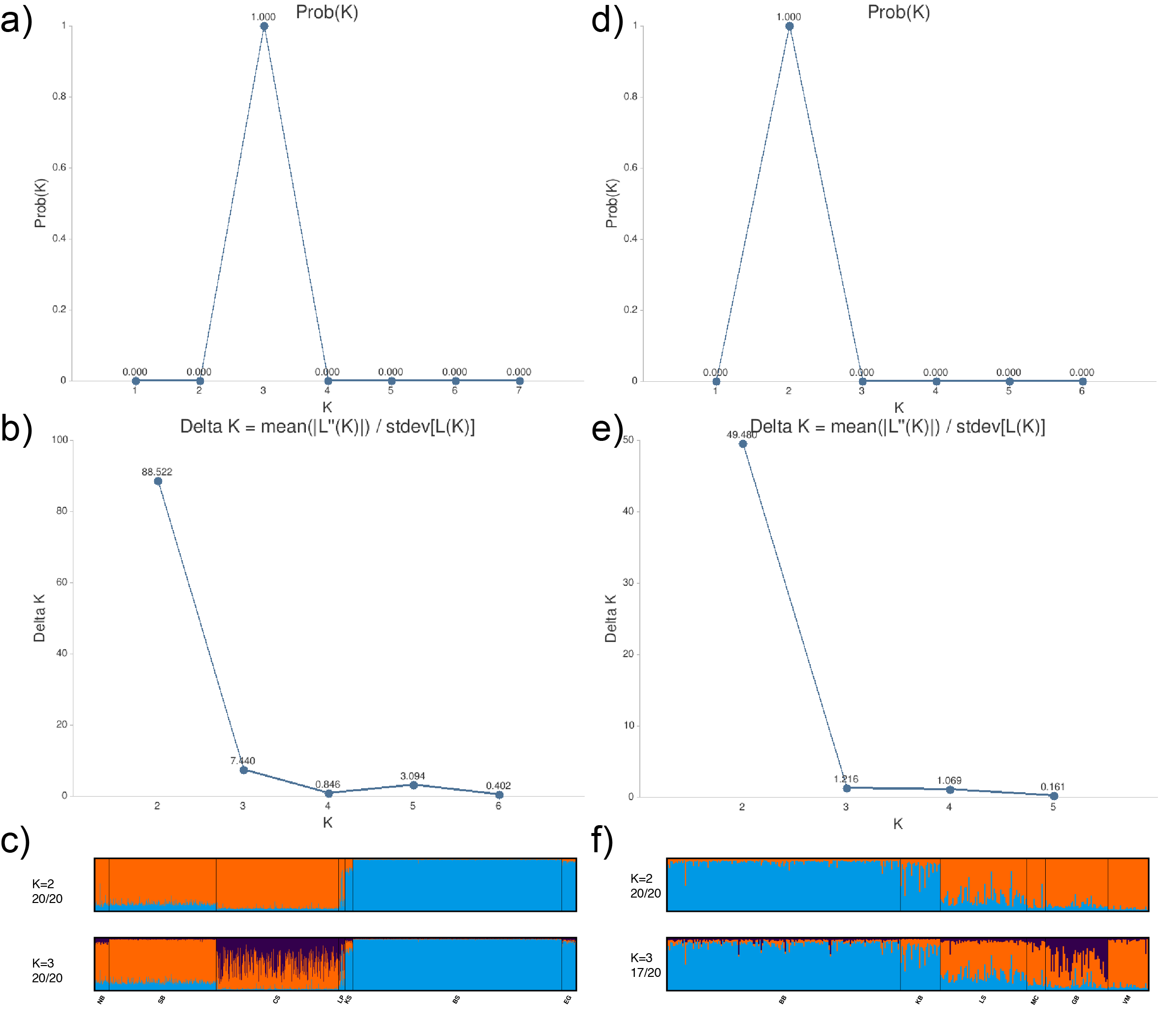
**

**Figure B.** CLUMPAK output for Structure runs using the LOCPRIOR=1 for the complete set of samples from: a–c) the Polar Basin cluster, and d–f) the Canadian Arctic Archipelago. a) and d) show the preferred number of clusters using the Pritchard method; b) and e) show the preferred number of clusters according to the Evanno method; c) and f) show the majority modes for the preferred number of clusters. Both show east–west differentiation in the preferred number of clusters. In f), the results for K=3 are also plotted (though they are not preferred by either method), as they show potential differentiation of the Gulf of Boothia from the neighbouring M’Clintock Channel management unit, as has previously been reported [6]. Note that Davis Strait samples have been excluded from the run for the Canadian Arctic Archipelago, because of their sheer number and because Davis Strait represents an admixture zone between the Archipelago and the Hudson Complex.

**Figure C.** Admixture plot produced by BAPS for *K*=6. A single individual in LP with four missing loci (displayed here in white) was placed in its own genetic cluster during mixture clustering and was removed prior to admixture analysis, leaving five major genetic clusters. Management unit abbreviations are as in Table 1 of the main paper.

**
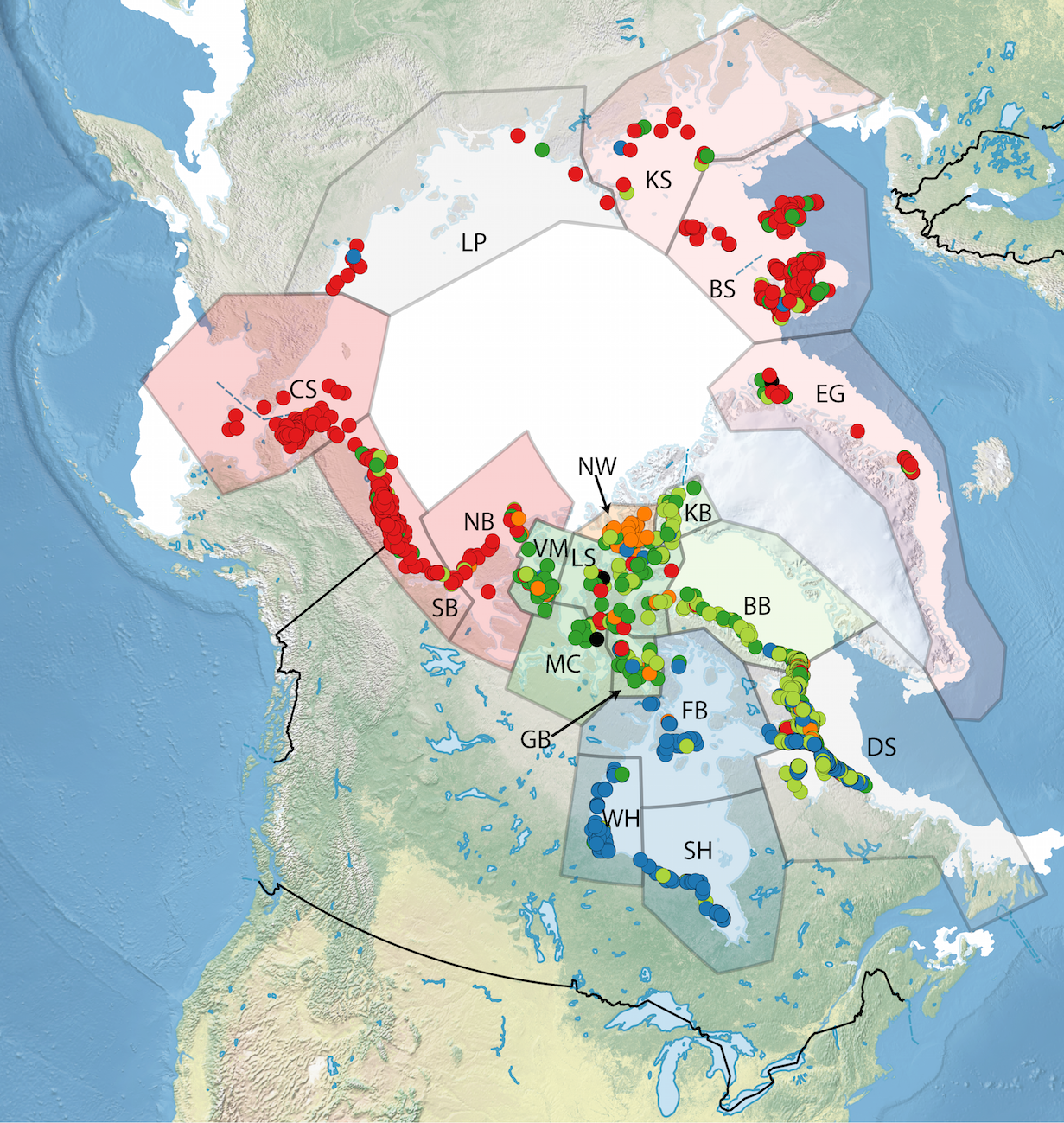
**

**Figure D.** Genetic cluster memberships for all individuals with microsatellite genotypes included in the original study of Peacock *et al.*, 2015. Individuals in Figure C who were “highly assigned” (i.e., posterior probability of non-admixture > 0.05) to one of the five major clusters identified by BAPS were retained as a training set with which to cluster all other individuals. Significantly admixed individuals are shown displayed in black. Management unit abbreviations are as in Table 1 of the main paper. As in Figure 6 of the main paper, sea ice extent during the breeding season is approximated using measurements for April 15, 2008. Colours are as in Figure 4 of the main manuscript.


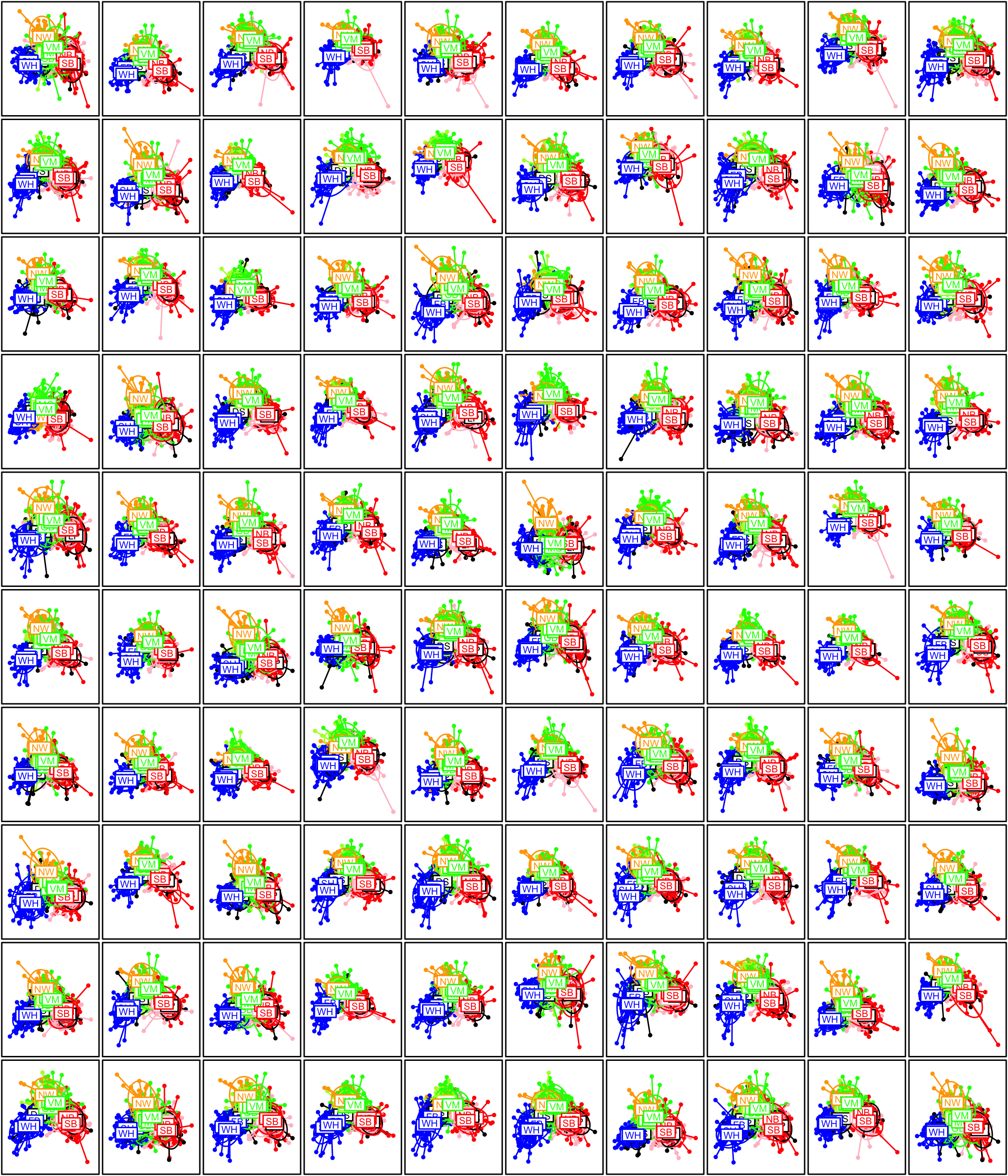


**Figure E.** Pollack plot showing PCAs (axes: x = PC1, y = PC2) of 100 independent random subsamples of ≤30 individuals per management unit from the complete 2748-individual polar bear dataset. Only individuals who were fully genotyped for the fourteen loci listed in the main paper were included. Management units are colour-coded as in the main manuscript. In cases where four major clusters cannot clearly be distinguished, they usually become apparent by viewing the third PC (Figure F).


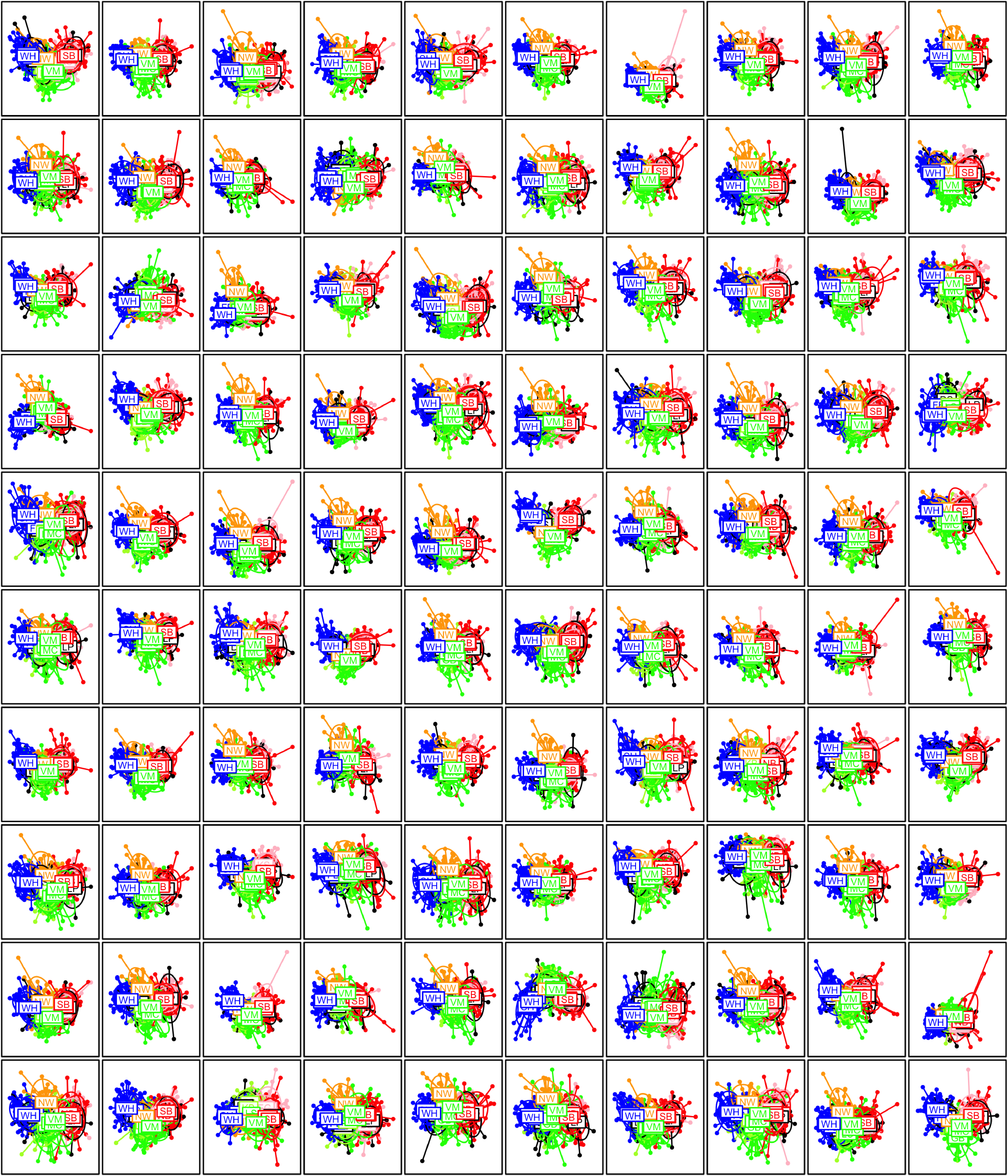


**Figure F.** Pollack plot showing PCAs (axes: x = PC1, y = PC3) of 100 independent random subsamples of ≤30 individuals per management unit from the complete 2748-individual polar bear dataset. These are the same random subsets as used in Figure E. Only individuals who were fully genotyped for the fourteen loci listed in the main paper were included. Management units are colour-coded as in the main manuscript.

**References**

1. Viengkone M. Population Structure and Space-use of Polar Bears (*Ursus maritimus*) in Hudson Bay [M.Sc. Thesis]. Edmonton, AB, Canada: University of Alberta; 2015.

2. IUCN/SSC Polar Bear Specialist Group. 2014 Polar Bear Status Table 2015 [updated Jan. 24, 2015; cited 2015 May 11, 2015]. Available from: <http://pbsg.npolar.no/en/status/status-table.html>.

3. Peacock E, Taylor MK, Laake J, Stirling I. Population ecology of polar bears in Davis Strait, Canada and Greenland. J Wildl Manage. 2013;77(3):463–76. doi: 10.1002/jwmg.489.

4. Paetkau D, Amstrup SC, Born EW, Calvert W, Derocher AE, Garner GW, et al. Genetic structure of the world's polar bear populations. Mol Ecol. 1999;8(10):1571–84. PubMed PMID: ISI:000083466800002.

5. Matishov GG, Chelintsev NG, Goryaev YI, Makarevich PR, Ishkulov DG. Assessment of the amount of polar bears (*Ursus maritimus*) on the basis of perennial vessel counts. Dokl Earth Sci. 2014;458(2):1312–6. doi: 10.1134/S1028334X14100298.

6. Campagna L, Van Coeverden de Groot PJ, Saunders BL, Atkinson SN, Weber DS, Dyck MG, et al. Extensive sampling of polar bears (*Ursus maritimus*) in the Northwest Passage (Canadian Arctic Archipelago) reveals population differentiation across multiple spatial and temporal scales. Ecology and Evolution. 2013;3(9):3152–65. doi: 10.1002/ece3.662.
